# An SR protein is essential for activating DNA repair in malaria parasites

**DOI:** 10.1242/jcs.258572

**Published:** 2021-08-17

**Authors:** Manish Goyal, Brajesh Kumar Singh, Karina Simantov, Yotam Kaufman, Shiri Eshar, Ron Dzikowski

**Affiliations:** Department of Microbiology & Molecular Genetics, The Kuvin Center for the Study of Infectious and Tropical Diseases, IMRIC, The Hebrew University-Hadassah Medical School, Jerusalem 91120, Israel

**Keywords:** Malaria, *Plasmodium falciparum*, SR protein, Splicing factor, DNA damage response, Artemisinin

## Abstract

*Plasmodium falciparum*, the parasite responsible for the deadliest form of human malaria, replicates within the erythrocytes of its host, where it encounters numerous pressures that cause extensive DNA damage, which must be repaired efficiently to ensure parasite survival. Malaria parasites, which have lost the non-homologous end joining (NHEJ) pathway for repairing DNA double-strand breaks, have evolved unique mechanisms that enable them to robustly maintain genome integrity under such harsh conditions. However, the nature of these adaptations is unknown. We show that a highly conserved RNA splicing factor, *P. falciparum* (*Pf*)SR1, plays an unexpected and crucial role in DNA repair in malaria parasites. Using an inducible and reversible system to manipulate *Pf*SR1 expression, we demonstrate that this protein is recruited to foci of DNA damage. Although loss of *Pf*SR1 does not impair parasite viability, the protein is essential for their recovery from DNA-damaging agents or exposure to artemisinin, the first-line antimalarial drug, demonstrating its necessity for DNA repair. These findings provide key insights into the evolution of DNA repair pathways in malaria parasites as well as the ability of the parasite to recover from antimalarial treatment.

## Abstract (precis)

**Summary:** There is an unexpected role for the alternative splicing factor *Pf*SR1 in activating the DNA damage response in the human malaria parasite *Plasmodium falciparum*.

## INTRODUCTION

Malaria remains one of the deadliest infectious diseases, killing approximately half a million people each year, primarily young children and pregnant women in sub-Saharan Africa (WHO, 2016). *Plasmodium falciparum* is the protozoan parasite responsible for over 90% of malaria deaths, and thus is considered the deadliest form of human malaria parasite. This parasite has a complex life cycle alternating between different biological niches in the human host and the Anopheline vector. Surprisingly, *Plasmodium* parasites have evolved this complex biology with ∼5700 protein coding genes, which is even lower than the gene number of the budding yeast *Saccharomyces cerevisiae*, which has a much simpler life cycle (Gardner et al., 2002). One way by which eukaryotes can expand their protein repertoire out of a limited gene number is through alternative splicing of their pre-mRNA transcripts. Alternative splicing is thought to be regulated by splicing factors that recognize specific splicing signals. Serine/arginine-rich (SR) proteins are known splicing factors that bind splicing enhancers in a sequence-specific manner. Several putative SR proteins have been annotated in the *P. falciparum* genome*,* but only *P. falciparum* (*Pf*)SR1 (PF3D7_0517300) has been shown to function as an alternative splicing factor *in vivo* (Eshar et al., 2012). In addition to alternative splicing, *Pf*SR1 regulates mRNA levels by binding to specific RNA motifs (Eshar et al., 2015). In recent years, it has become clear that RNA processing is linked to the DNA damage response (DDR), and several splicing factors have been implicated as gatekeepers of genome stability (Naro et al., 2015; Shkreta and Chabot, 2015).

Malaria symptoms appear when *Plasmodium* parasites invade and replicate within human red blood cells, where they are exposed to numerous sources that challenge their genome stability. The blood stage forms of malaria parasites uniquely replicate by consecutive mitoses during schizogony, and are particularly prone to replication errors. In addition, they live in a highly oxygenated environment and produce potent DNA-damaging agents while digesting hemoglobin. Furthermore, the parasites are also exposed to oxidative substances released from immune cells as response to infection, as well as to reactive oxygen species (ROS) induced by chemotherapeutic agents used to treat malaria (Nathan and Shiloh, 2000; Gupta et al., 2016). However, the mechanisms by which the parasite maintains its genome integrity under such extreme conditions are poorly understood.

Interestingly, *Plasmodium* parasites appear to be more resistant to DNA damage than most eukaryotes (Calhoun et al., 2017), despite the evolutionary loss of non-homologous end joining (NHEJ), a primary double-strand break (DSB) repair pathway. In addition, blood stage parasites are haploids, therefore lacking the substrates necessary for homologous recombination (HR). Instead they have been shown to use an alternative non-homologous end joining (A-NHEJ) pathway to repair DSBs (Kirkman et al., 2014; Singer et al., 2015). In addition to these mechanisms, recent *in silico* analysis identified components of the DNA mismatch repair pathway in the *P. falciparum* genome (Tarique et al., 2017), which were linked to generating genetic diversity and drug resistance (Ahmad and Tuteja, 2014; Bethke et al., 2007; Castellini et al., 2011). However, the molecular basis for the robust DNA repair in blood stage parasites, in the absence of NHEJ, remains almost entirely unknown.

Here, we show that *Pf*SR1 interacts with proteins involved in different processes of RNA metabolism as well as the DDR. We demonstrate that *Pf*SR1 is recruited to the site of DNA damage where it interacts with the phosphorylated core histone *Pf*H2A (γ-*Pf*H2A). Furthermore, by creating an inducible knockdown system for the endogenous *Pf*SR1, we revealed that *Pf*SR1 is essential for the ability of the parasite to activate the DDR to overcome DNA damage and exposure to artemisinin.

## RESULTS

### Stage-dependent analysis of the *Pf*SR1 interactome points towards its involvement in multiple processes of RNA metabolism and the DDR

**Fig. 1.**
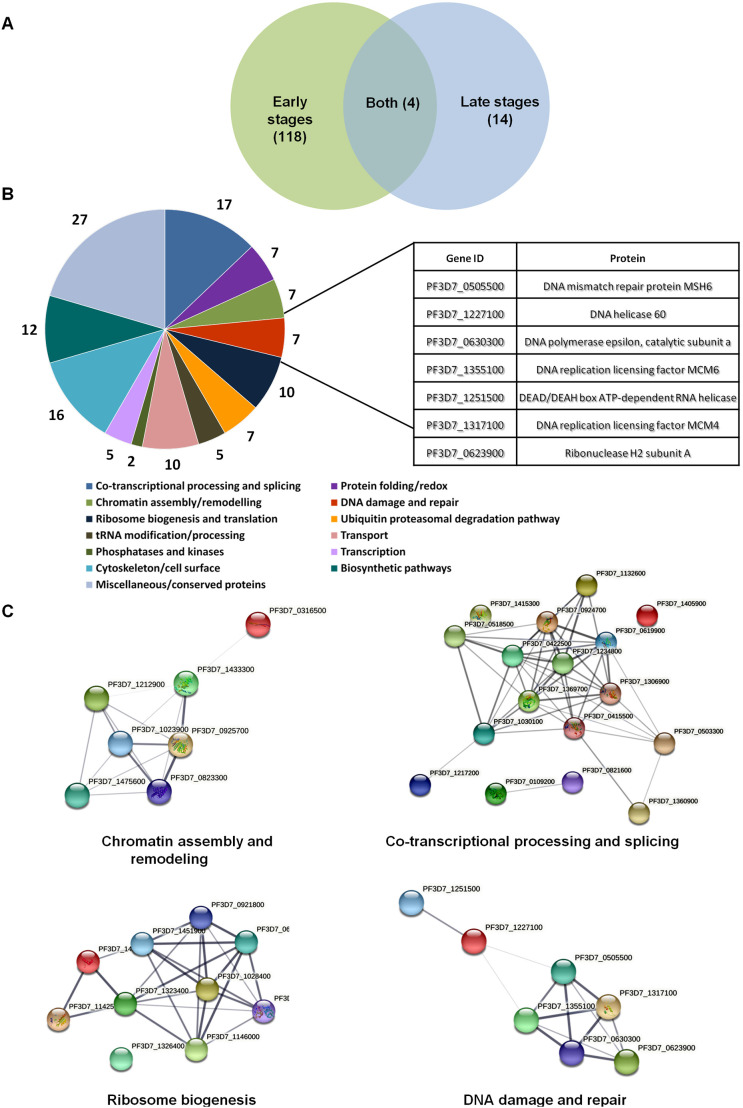
**The *Pf*SR1 interactome is stage dependent and enriched with proteins involved in several processes of RNA metabolism and DNA damage repair.** (A) A Venn diagram of stage-dependent *Pf*SR1 interactome showing that most interactions (118 proteins) occur during the first 20 h of IDC and only a few (14 proteins) at late stages of IDC. (B) Pie chart of putative gene annotation of the proteins predicted to be involved in RNA metabolism and DNA damage repair that were specifically enriched in the *Pf*SR1–Halo pulldown parasites versus control Halo pulldown parasites. The number of proteins in each group is presented and the details of DNA repair proteins are shown in the table on the right. Gene annotations were derived from PlasmoDB using annotated GO process. (C). Protein-protein interaction networks of *Pf*SR1 interacting proteins. Interaction networks among proteins of different functional groups were calculated using the STRING database. Line thickness indicates the probability of the predicted interactions.

We have previously shown that *Pf*SR1 regulates alternative splicing and RNA levels in *P. falciparum* (Eshar et al., 2012, 2015)*.* To better understand the mechanisms by which *Pf*SR1 functions as a regulator of gene expression in *P. falciparum*, we were interested in identifying its interacting proteins throughout its intra-erythrocytic development (IDC). We transfected NF54 parasites with an expression vector that allows a fine-tuned overexpression of *Pf*SR1 fused with a Halo tag at the N-terminus (Fig. S1A). This episomal system allowed us to perform gradual overexpression of *Pf*SR1 by increasing blasticidin concentrations (Eshar et al., 2012), and to perform highly specific pull-down assays of *Pf*SR1-interacting proteins *in vivo* (England et al., 2015). As a first step, we gradually over expressed Halo*–Pf*SR1 by selecting on increasing blasticidin concentrations (2, 6 and 10 µg/ml) and determined the optimal selection pressure needed to detect episomal expression (6 µg/ml; Fig. S1B). We then performed stage-dependent pull-down assays on early and late stage parasites expressing either Halo–*Pf*SR1 or a mock plasmid expressing only the Halo tag (Fig. S1C,D). To identify proteins that were significantly enriched in the fractions recovered from Halo*–Pf*SR1, we performed proteomic analysis on both parasite populations by liquid chromatography followed by tandem mass spectrometry (LC MS/MS). Proteins that were enriched at least 8-fold in the Halo*–Pf*SR1 parasites in two out of three biological replicates are listed in Table S1. Remarkably, it appears that most of the interactions of *Pf*SR1 with other proteins occur at the early stages of the IDC of the parasites (20 h post invasion, hpi) and only a few occur at late stages (36 hpi; Fig. 1A). We found that *Pf*SR1 interacts with proteins predicted to function at several levels of RNA metabolism, from transcription and chromatin organization, through splicing and maturation, to translation (Fig. 1B). Furthermore, *Pf*SR1 also interacts with several proteins implicated in the DDR. We further used computational analysis, using the string database (https://string-db.org/) to predict the protein–protein interaction networks among *Pf*SR1-interacting partners based on known and predicted structure and function of these proteins (Fig. 1C). This analysis, which predicts possible interactions among proteins involved in chromatin organization, transcription and splicing, ribosome biogenesis and DDR, provides an additional indication that *Pf*SR1 may play a role in these processes in addition to its role in constitutive and alternative splicing.

### *Pf*SR1 expression is required for the ability of the parasite to recover from DNA damage

**Fig. 2.**
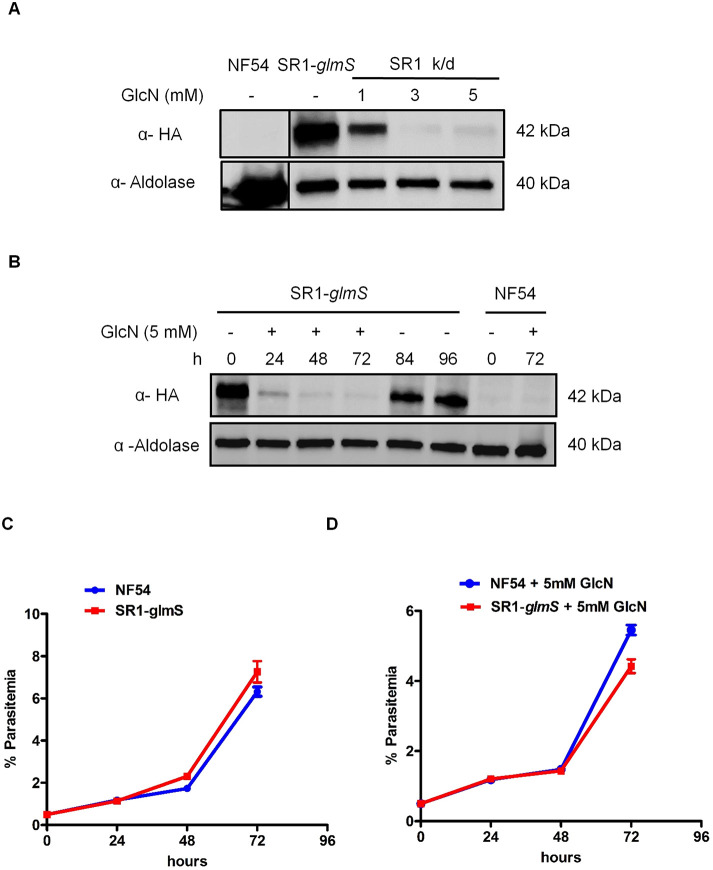
**Inducible knockdown of endogenous *Pf*SR1.** (A) Increasing concentrations of GlcN for 72 h cause decreased expression of endogenous *Pf*SR1. (B) Time-dependent depletion of *Pf*SR1 in parasites growing on 5 mM GlcN over a 72 h time course, which could be reversed by removing GlcN. (C) *Pf*SR1-*glmS* and NF54 parasite lines have a similar growth on regular medium. (D) Parasites in which *Pf*SR1 was knocked down have a slight delayed growth rate compared with parental NF54 line. Results in C,D are mean±s.e.m. All experiments were performed in three independent biological replicates.

SR proteins have been implicated in several processes of RNA metabolism, but very little is known regarding their possible role in DDR. Intrigued by the interactions of *Pf*SR1 with proteins predicted to be involved in the DDR (Fig. 1; Table S1), we decided to investigate its function in this process. Towards this aim, we used the CRISPR/Cas9 system to create a transgenic line in which the 3′ terminus of the endogenous open reading frame is replaced and fused with an HA epitope tag and the *glmS* ribozyme (Fig. S2). This parasite line enables the quantification and visualization of the endogenous *Pf*SR1 as well as to induce expression knockdown by adding glucosamine (GlcN) to the culture medium as described previously (Prommana et al., 2013). After isolating a clonal population of the transgenic line, we determined that incubation of the parasites with 5 mM GlcN for 72 h results in an almost complete knockdown of *Pf*SR1 expression (Fig. 2A). Furthermore, we observed that with 5 mM GlcN, *Pf*SR1 expression decreases over time and is reversible upon GlcN removal from the culture medium (Fig. 2B). Under normal growth conditions, the *Pf*SR1-*glmS* parasite line grew at a similar rate to the NF54 wild-type population; however, when *Pf*SR1 was knocked down by GlcN it showed a slight reduction in its growth rate, which is likely multifactorial (Fig. 2C,D).

**Fig. 3.**
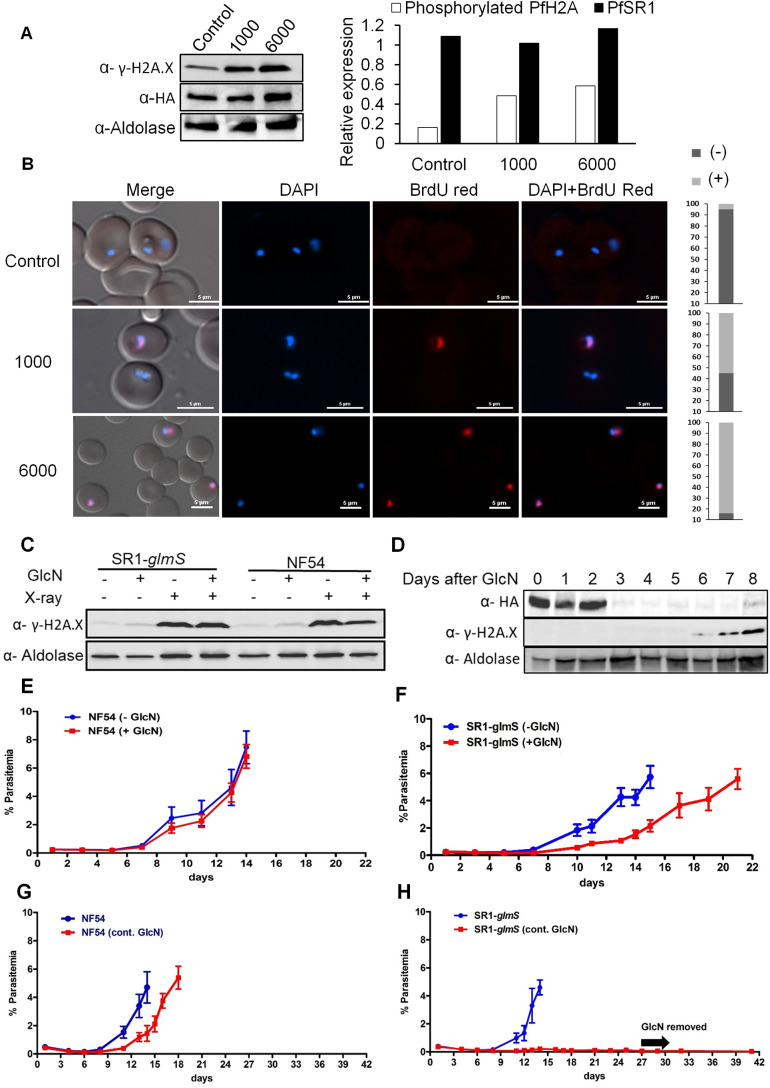
***Pf*SR1 is essential for parasite recovery from DNA damage caused by X-ray irradiation.** (A) Exposure of *Pf*SR1-*glmS* parasite lines to increasing levels of X-ray irradiation is associated with increased levels of γ-*Pf*H2A. (B) DNA fragmentation imaging by TUNEL assay of parasites exposed to increasing levels of X-ray irradiation demonstrating increased levels of DNA breaks. Quantification of the percentage of TUNEL-positive and -negative nuclei in each treatment is presented on the right (*n*=100). Scale bars: 5 µm. (C) Western blot analysis demonstrating that the levels of γ-*Pf*H2A are elevated 15 min after X-ray irradiation regardless of GlcN treatment or parasite line (5 mM for 72 h prior to irradiation). (D) Western blot analysis of *Pf*SR1-*glmS* parasite line growing on GlcN over time, indicating that γ-*Pf*H2A accumulates ∼1 week after *Pf*SR1 downregulation. (E) Growth curves of wild-type NF54 parasites exposed to a near lethal dose of X-ray irradiation of 6000 rad. Parasites were grown either on regular medium (− GlcN) or with medium supplemented with 5 mM GlcN for 72 h and washed immediately before irradiation (+ GlcN). (F) Growth curves of *Pf*SR1-*glmS* parasites exposed to 6000 rad X-ray irradiation and grown either on regular medium (− GlcN) or with medium supplemented with 5 mM GlcN for 72 h and washed immediately before irradiation (+GlcN). (G) Growth curves of wild-type NF54 parasites exposed to 6000 rad X-ray irradiation and grown either on medium supplemented continuously with 5 mM GlcN (cont. GlcN) or on regular medium. (H) Growth curves of *Pf*SR1-*glmS* parasites exposed to 6000 rad X-ray irradiation and grown either in media supplemented continuously with 5 mM GlcN (cont. GlcN) or in regular media. Each of the curves represents the average parasitemia of three biological replicates at each timepoint. Error bars represent s.e.m. Western blots shown are representative of three experiments. Densitometry analysis was performed using ImageJ software.

We then used this transgenic line to determine whether *Pf*SR1 is involved in the DDR. In yeast and mammals, a DSB triggers the DDR, which rapidly leads to phosphorylation of the core histone isoform H2A.X to form γ-H2A.X, which marks the site of damaged DNA (Rogakou et al., 1998). We have recently demonstrated that in *P. falciparum* that lacks the H2A.X variant, the canonical *Pf*H2A (PF3D7_0617800) is phosphorylated on S121 upon exposure to sources of DNA damage, and that phosphorylated *Pf*H2A is recruited to foci of damaged chromatin shortly after exposure to sources of damage (Goyal et al., 2021). This ability to specifically detect the dynamics of *Pf*H2A phosphorylation using an anti γ-H2A.X antibody provides a useful marker for studying the DDR in *P. falciparum* (Goyal et al., 2021). As a first step, we were interested to confirm that DNA damage could be detected in our transgenic lines in a similar manner to what we have previously shown in NF54 parasites. We exposed the *Pf*SR1-*glmS* transgenic line to different doses of X-ray irradiation (1000 and 6000 rad), and found that the levels of γ-*Pf*H2A gradually increased following the irradiation of the parasites; however, the levels of *Pf*SR1 expression were not significantly elevated (Fig. 3A). To further demonstrate direct measurement of DNA damage in response to increased levels of X-ray irradiation, we performed a TUNEL assay and visualized DNA fragmentation in the nuclei of the parasites. We clearly show that in untreated parasites less than 10% had a signal; however, in parasites exposed to 1000 and 6000 rad, a strong signal was observed in over 50% and 90% of the nuclei, respectively (Fig. 3B). These data demonstrate that X-ray irradiation induces DNA damage in non-replicating ring stage *Pf*SR1-*glmS* parasites and that γ-*Pf*H2A levels are elevated during DDR. Interestingly, in both *Pf*SR1-*glmS* and NF54 parasites, we observed an increase in γ-*Pf*H2A levels 15 min following X-ray irradiation (6000 rad), irrespective of GlcN treatment (Fig. 3C). We then tested whether downregulation of *Pf*SR1 expression was associated with DDR *in vivo* over a longer period of time, and found that the levels of γ-*Pf*H2A were elevated ∼1 week following *Pf*SR1 knockdown (Fig. 3D), while only basal levels of γ-*Pf*H2A were observed in NF54 parasites (Fig. S3).

To determine whether *Pf*SR1 is essential for the ability of the parasite to recover from DNA damage, we irradiated parasites with lethal (6000 rad) X-ray doses and followed their rate of recovery as previously described (Calhoun et al., 2017). We found that NF54 parasites recover ∼13 days after X-ray irradiation, and that pre-incubation with GlcN for 72 h prior to irradiation did not affect their growth rate (Fig. 3E). However, in the *Pf*SR1-*glmS* line, a 5–6-day delay in parasite recovery was observed following *Pf*SR1 knockdown for 72 h prior to irradiation as compared to parasites that were not pre-incubated with GlcN (Fig. 3F). In this set of experiments, GlcN was removed from the culture medium just before irradiation, thus allowing the parasites (in which *Pf*SR1 was knocked down) to express *Pf*SR1 while recovering from the damage caused by X-ray irradiation. To determine whether *Pf*SR1 is indeed essential for the recovery of parasites from X-ray irradiation, we performed similar experiments, only this time we compared the recovery of parasites in the presence of GlcN over the entire course of the experiment (i.e. under continuous knockdown of *Pf*SR1) even after X-ray irradiation. Strikingly, while NF54 parasites recovered on continuous growth with GlcN (Fig. 3G), *Pf*SR1-*glmS* parasites could not recover from the damage caused by the lethal dose of irradiation unless *Pf*SR1 was expressed (Fig. 3H). To exclude the possibility that the observed phenotype is due to accumulated growth defects of long-term *Pf*SR1 depletion, we show that *Pf*SR1-depleted line maintains their delayed growth rate constantly over a long period of time without irradiation (Fig. S4). Altogether, these data indicate that *Pf*SR1 expression is essential for *P. falciparum* parasites to overcome the damage caused by X-ray irradiation and points towards its involvement in the DDR.

### *Pf*SR1 is essential for repairing DNA damage

**Fig. 4.**
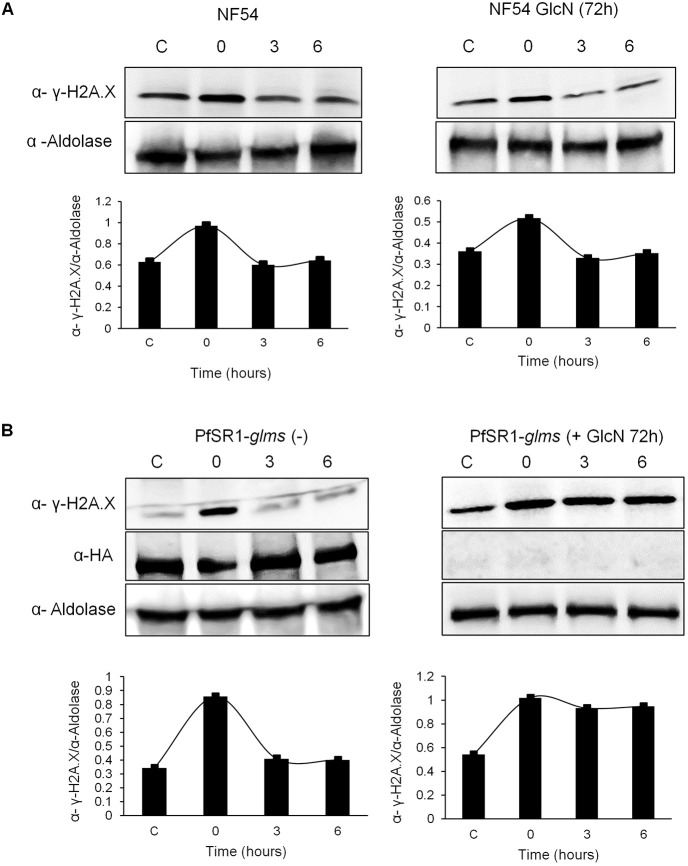
***Pf*SR1 is essential for repairing damaged DNA.** (A) Upper panels, western blot analysis of proteins extracted at 15 min, 3 h and 6 h (time 0, 3 and 6, respectively) following exposure to a sublethal dose of X-ray irradiation (1000 rad). Protein was extracted from tightly synchronized ring stage NF54 parasites growing either on regular medium (left) or on medium supplemented with 5 mM GlcN (right). Lower panels, densitometry quantification of the ratio between the western blot signals obtained for γ-*Pf*H2A and aldolase. These analyses demonstrate that the level of γ-*Pf*H2A, which increases 15 min after irradiation, is reduced 3 h later regardless of GlcN (B). Upper panels, western blot analysis of protein extracted 15 min, 3 h and 6 h (time 0, 3 and 6, respectively) following exposure to a sublethal dose of X-ray irradiation (1000 rad). Protein was extracted from tightly synchronized ring stage *Pf*SR1-*glmS* parasites growing either on regular medium (left) or on medium supplemented with 5 mM GlcN (right). Lower panels, densitometry quantification of the ratio between the western blot signals obtained for γ-*Pf*H2A and aldolase. These analyses demonstrate that in the *Pf*SR1-*glmS* parasites DNA damage could only be repaired in parasites growing on regular media and not those growing on GlcN in which *Pf*SR1 is downregulated. Densitometry analysis was performed using ImageJ software for the western blots shown, which are representative of three experiments. C (control), before irradiation.

The association of *Pf*SR1 with damaged chromatin and its essentiality for the ability of the parasites to recover from exposure to X-ray irradiation led us to test its involvement in DNA damage repair. We have recently shown that *Pf*H2A phosphorylation is dynamic and, over time, as the parasite activates the repair machinery this phosphorylation is removed. Thus, these phosphorylation dynamics were used to establish a direct DNA repair assay in *P. falciparum* (Goyal et al., 2021). To exclude the possible effect of GlcN on the ability of the parasite to activate the DNA repair machinery, NF54 parasites were exposed to sub-lethal levels of X-ray irradiation (1000 rad), and put back in culture to allow the parasites to repair the damaged DNA (Fig. 4A). We observed that in NF54 parasites growing in either regular medium (left) or in medium supplemented with 5 mM GlcN (right), the levels of γ-*Pf*H2A increased 15 min after irradiation. However, 3 and 6 h post irradiation, the levels of γ-*Pf*H2A decreased back to the basal level, indicating that the DNA repair process was initiated (Fig. 4A). We then performed the same assay on the *Pf*SR1-*glms* transgenic line. We found that when *Pf*SR1-*glms* parasites grow in regular medium and express *Pf*SR1 (Fig. 4B, left panel), they were able to activate the DNA repair machinery similar to what is seen in NF54 parasites. In marked contrast, when *Pf*SR1 expression was downregulated by GlcN, the levels of γ-*Pf*H2A remained constant, similar to the levels measured immediately after irradiation (Fig. 4B, right panel). These data indicate that in the absence of *Pf*SR1 expression, *P. falciparum* parasites are impaired in their ability to repair DNA damage.

### *Pf*SR1 is recruited to the sites of DNA damage

**Fig. 5.**
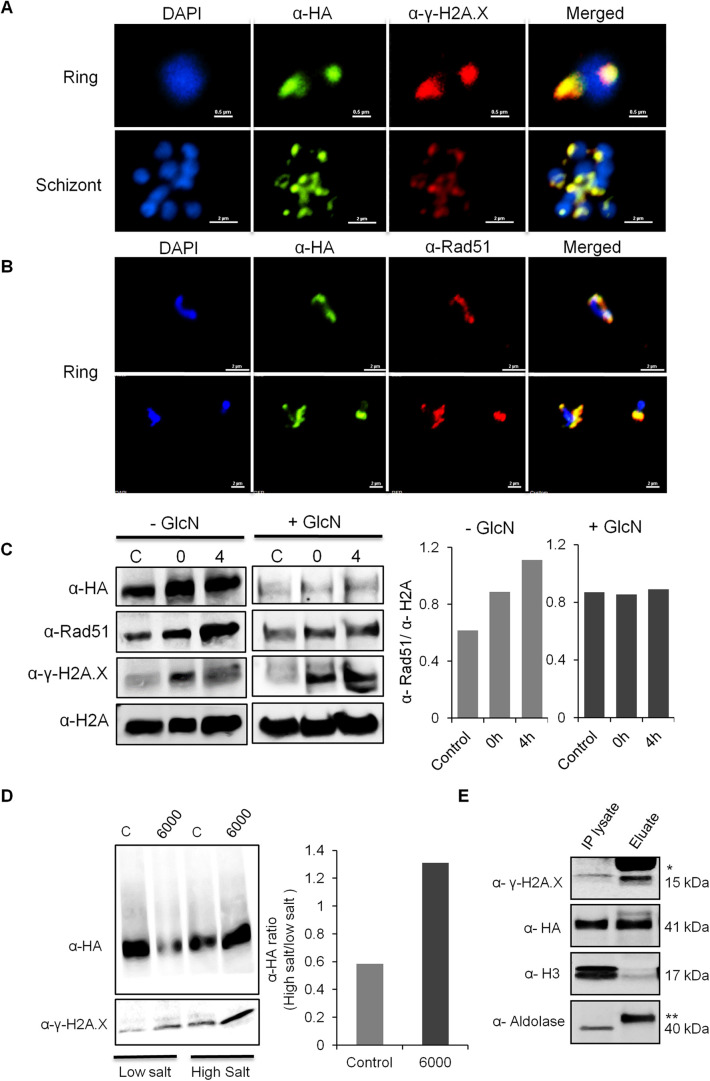
***Pf*SR1 is recruited to the site of damaged DNA and interacts with phosphorylated H2A.** (A) Immunofluorescence assay demonstrating that *Pf*SR1 (green) is associated with γ-*Pf*H2A (red) in the nucleus of early (upper panel) and late stage (lower panel) *Pf*SR1-*glmS* parasites 15 min after X-ray irradiation. (B) Immunofluorescence assay demonstrating that *Pf*SR1 (green) colocalizes with *Pf*Rad51 (red) in the nucleus of ring stage *Pf*SR1-*glmS* parasites after X-ray irradiation. DNA is stained with DAPI. Scale bars: 2 µm. (C) *Pf*SR1 expression affects nuclear accumulation of *Pf*Rad51 following exposure to X-ray irradiation. Nuclear levels of *Pf*Rad51 were detected by western blotting in *Pf*SR1-*glmS* parasites growing in the presence (left) or absence (right) of 5 mM GlcN. Nuclei were isolated from parasites before irradiation (denoted by C, control), immediately after (0 h) and 4 h (4 h) post irradiation (3000 rad). Densitometry analysis indicate that nuclear accumulation of *Pf*Rad51 4 h post irradiation is detected in only in *Pf*SR1-expressing parasites (right panel). (D) *Pf*SR1 association with chromatin is elevated following exposure to irradiation. Nuclear proteins were extracted from *Pf*SR1-*glmS* parasites under low and high salt conditions, before and after exposure to irradiation. Western blot analysis using anti-HA (upper) and anti-γ-H2A.X antibody (lower) detected both *Pf*SR1 and γ-*Pf*H2A. *Pf*SR1 is significantly enriched in the high salt extract fraction when compared to its levels in the low salt fraction, following X-ray irradiation. Densitometry analysis of the ratio of *Pf*SR1 in high salt to in low salt is shown on the right demonstrating 2-fold enrichment. (E) *Pf*SR1 interacts with γ-*Pf*H2A as detected by co-IP experiment using anti-γ-H2A.X antibody. Western blot analysis for the presence of γ-*Pf*H2A, *Pf*SR1 (α HA), core histone H3 (α H3), and cytosolic aldolase in the input (IP lysate) and the pulldown fraction (eluate) demonstrate that *Pf*SR1 was pulled down with the anti γ-H2A.X antibodies. Light chain of primary antibody is marked with * and heavy chain is marked with **. Images in A and B are representative of at least three experiments. Western blots shown are representative of three experiments. Densitometry analysis was performed using ImageJ software.

The fact that the γ-*Pf*H2A is recruited to damaged chromatin shortly after exposure to sources of DNA damage (Goyal et al., 2021) enabled us to visualize a possible association between *Pf*SR1 and the nuclear site of DNA damage. We used the transgenic *Pf*SR1-*glmS* line to visualize the HA-tagged endogenous *Pf*SR1 and determined whether it colocalizes with γ-*Pf*H2A. We found a strong association (78/83 independently counted nuclei) between *Pf*SR1 and the site of DNA damage in both early- and late-stage parasites (Fig. 5A). In addition, we found that in irradiated ring-stage parasites *Pf*SR1 colocalized with *Pf*Rad51 (Fig. 5B, 39/50 independently counted nuclei), providing additional support for its recruitment to the site of DNA damage. Interestingly, we observed that in parasites that express *Pf*SR1, that the γ-*Pf*H2A signal disappears 4 h post irradiation in parallel to the accumulation of *Pf*Rad51 in the nucleus. However, in parasites in which *Pf*SR1 expression is knocked down, *Pf*Rad51 does not accumulate in the nucleus and thus the levels of γ-*Pf*H2A do not decrease over time (Figs S5, S6). To provide further support for these visual observations we measured *Pf*Rad51 and γ-*Pf*H2A protein levels before and after irradiation in isolated nuclei of parasites that either expressed *Pf*SR1 or those in which *Pf*SR1 was knocked down. We found that *Pf*Rad51 levels increased and γ-*Pf*H2A decreased at 4 h post irradiation only in parasites in which *Pf*SR1 expression was not perturbed (Fig. 5C). Next, we measured *Pf*SR1 association with chromatin by differential salt fractionation of nuclei, which separates proteins that are strongly bound to chromatin by high salt extraction (see Materials and Methods) before and after X-ray irradiation (6000 rad). We found that the proportion of chromatin-bound *Pf*SR1 increases after exposure to irradiation, when compared with the level in control untreated parasite nuclei (Fig. 5D). Encouraged by this observation, we were interested to determine whether *Pf*SR1 interacts with the phosphorylated *Pf*H2A at the damaged loci. To this end, we used the anti-γ-H2A.X antibody to perform co-immunoprecipitation (co-IP) on nuclear extracts of irradiated parasites. Western blot analysis of this co-IP experiment shows that both γ-*Pf*H2A and *Pf*SR1 were present in the IP eluted fraction (Fig. 5E), indicating that they were co-immunoprecipitated. Moreover, as expected, only a small amount (compared with the input) of the core histone H3 was also pulled down, and the cytoplasmic aldolase could not be detected. These data indicate that *Pf*SR1 is recruited to the site of DNA damage where it interacts with γ-*Pf*H2A and appears to be essential for activating DDR.

### *Pf*SR1 is essential for the ability of the parasite to recover from artemisinin exposure

**Fig. 6.**
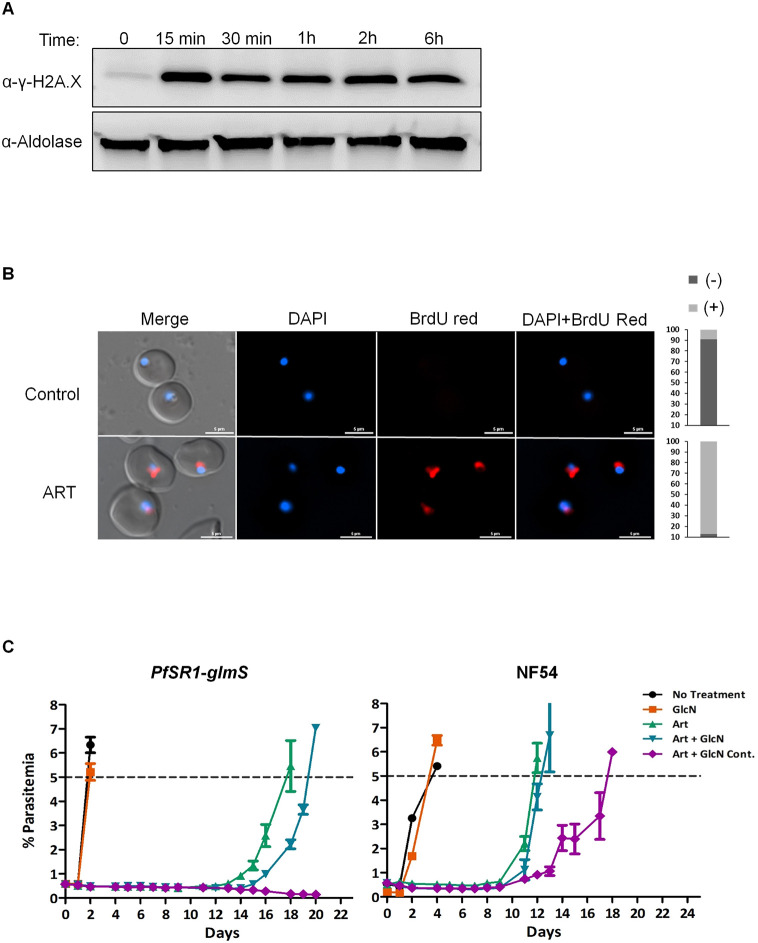
***Pf*SR1 expression is required to overcome artesunate exposure.** (A) Western blot analysis demonstrating the increase in γ-*Pf*H2A levels following exposure of tightly synchronized ring stage parasites to 700 nM artesunate for different time periods. Western blot shown is representative of three experiments. (B) DNA fragmentation imaging by TUNEL assay of parasites exposed to 700 nM artesunate for 15 min, demonstrating increased levels of DNA breaks. Quantification of the ratio of TUNEL positive and negative nuclei in each treatment is presented on the right (*n*=100). Scale bars: 5 µm. (C) Growth curves of tightly synchronized ring stage *Pf*SR1-*glmS* (left) and NF54 parasites (right), treated with 700 nM artesunate for 6 h. GlcN was either not added (Art, green triangle), added 72 h prior to artesunate treatment (Art+GlcN, blue triangle), or added continuously throughout the entire recovery period (Art+GlcN cont., purple diamond). Parasites growing on regular medium without any treatment (no treatment, black circle) and parasites treated only with GlcN (GlcN, orange square) are presented as controls. The results presented are the average parasitemia of three biological replicates measured daily. Error bar represents s.e.m.

The cytotoxic effect of artemisinin, the first-line antimalarial chemotherapeutic, was recently attributed to its ability to cause DNA damage mediated by ROS (Gopalakrishnan and Kumar, 2015). Moreover, a short exposure of parasites to artemisinin elicits a DDR that is similar to that induced by the alkylating agent methyl methanesulphonate (MMS), which further supports the idea that artemisinin acts as a DNA-damaging agent (Gupta et al., 2016). We found that the levels of γ-*Pf*H2A are elevated following exposure of tightly synchronized ring stage parasites to artesunate (ART), the semisynthetic derivative of artemisinin (Fig. 6A), and that by 15 min after artesunate treatment, DNA fragmentation could be visualized by TUNEL in more than 95% of the parasites (Fig. 6B). Considering the critical role that *Pf*SR1 plays in *P. falciparum* DDR, we tested whether it is required for parasite recovery from artemisinin pressure. To this end, we used the *Pf*SR1-*glmS* parasites and compared their ability to resist treatment with ART, when the endogenous *Pf*SR1 was expressed or when it was knocked down by GlcN incubation 72 h prior to ART treatment. Tightly synchronized ring stage parasites were incubated with 700 nM ART for 6 h, then washed and put back into culture on regular medium as previously described (Ghorbal et al., 2014). To evaluate the recovery rate of each parasite population from ART exposure, the level of parasitemia was measured daily by flow cytometry. We found that when *Pf*SR1 was expressed, parasites recovered ∼18 days after ART treatment, while the recovery of parasites in which *Pf*SR1 was knocked down prior to ART treatment was delayed (Fig. 6C). Strikingly, parasites in which *Pf*SR1 was continuously downregulated by the addition of GlcN to the medium were unable to recover from ART treatment, and no parasites were detected even after 21 days. These results imply that *Pf*SR1 expression is essential for parasite recovery from ART treatment.

## DISCUSSION

All eukaryotic organisms must maintain their genomic integrity while continuously exposed to challenges that threaten to damage their DNA (Naro et al., 2015). *Plasmodium* parasites, which replicate by schizogony within human red blood cells, are particularly prone to the most common sources causing DNA damage, such as replication defects and oxidative stress. In recent years, RNA-binding proteins (RBPs) have been implicated as important keepers of genome stability and as active players in the DDR (Dutertre et al., 2014; Naro et al., 2015; Shkreta and Chabot, 2015). Among these are several splicing factors, which in addition to their canonical role in splicing regulation, have been shown to play a direct role in preventing DNA damage during transcription and cellular segregation, as well as to be actively involved in DNA repair (Chan et al., 2014; Naro et al., 2015). In the current study, using a Halo tag episomal system, we found that, under normal growth conditions, the interactome of *Pf*SR1, an alternative splicing factor of *P. falciparum*, includes putative proteins that could be implicated in the DDR*.* These findings led us to hypothesize that, in addition to its role in regulating splicing and RNA metabolism, *Pf*SR1 is also involved in the DDR. Using endogenously tagged regulatable *Pf*SR1, we demonstrated that *Pf*SR1 is recruited to foci of damaged DNA where it interacts with γ-*Pf*H2A, a marker for damaged DNA. Furthermore, we showed that *Pf*SR1 is essential for *Pf*Rad51 accumulation in the nucleus of the parasite and for its ability to activate the DNA damage repair mechanism. In addition, we found that *Pf*SR1 is needed to overcome induced DNA damage following exposure to artemisinin, the first-line antimalarial drug.

At different stages of their life cycle, *Plasmodium* parasites replicate their haploid genome multiple times through consecutive mitosis cycles called schizogony, which makes them particularly prone to error during DNA replication. In addition, blood stage parasites live in a highly oxygenated environment, and while digesting large amounts of hemoglobin in their food vacuole they produce heme and hydroxyl radicals, which are potent DNA-damaging agents (Atamna and Ginsburg, 1993). Therefore, it is reasonable that the parasite has evolved efficient mechanisms to protect its genome integrity, which will allow it to proliferate in such conditions. Although little is known on DDR in *Plasmodium* parasites, their genome contains orthologs to many of the proteins involved in homologous recombination (HR), microhomology-mediated end joining (MMEJ) (Lee et al., 2014) and mismatch repair machinery (Tarique et al., 2017). Surprisingly, it appears that none of the components of the canonical non-homologous end joining (C-NHEJ) pathway are present in these parasites. Kirkman et al. created a transgenic line containing a system for inducing targeted DSBs in *P. falciparum.* They showed that malaria parasites utilize both HR and an alternative end joining pathway to maintain genome integrity, with preference for the alternative end-joining pathway as a primary method for repairing DSBs in the absence of heteroallelic homologous sequences that can serve as templates for HR (Kirkman et al., 2014).

In addition to the intrinsic metabolic oxidative stress sources, *Plasmodium* parasites are exposed to oxidative substances released from immune cells as a response to the infection, as well as to ROS induced by chemotherapeutic agents used to treat malaria, which can significantly disrupt genomic integrity (Gopalakrishnan and Kumar, 2015; Percário et al., 2012; Radfar et al., 2008). In this regard, activated artemisinin and its derivatives react promiscuously with nucleophile-harboring cellular components, leading to alkylation of DNA and proteins, which may cause protein misfolding and DNA damage (Tilley et al., 2016). Indeed, the recent association of the K13 mutation with artemisinin resistance was also accompanied by evidence for an enhanced unfolded protein response (UPR) in the resistant mutant lines (Mok et al., 2015). In addition, artesunate has been shown to induce oxidative DNA damage and a response in mammalian cancer cells (Berdelle et al., 2011; Kadioglu et al., 2017; Li et al., 2008), as well as DNA damage in malaria parasites, which is mediated by ROS (Gopalakrishnan and Kumar, 2015). Recently, it was demonstrated that exposure of *P. falciparum* to artemisinin induced the DDR, involving both transcriptional and epigenetic changes that are similar to what occurs when the parasite is exposed to the DNA-damaging agent MMS (Gupta et al., 2016). It is therefore likely that the cytotoxicity of artemisinin to malaria parasites involves direct DNA damage. The novel function of *Pf*SR1 in protecting the parasites from DNA damage and its critical role in enabling the parasite to overcome exposure to artemisinin, supports reported evidence that artemisinin and its derivatives damage the genome integrity of *Plasmodium* parasites. However, one cannot rule out that, in addition to its role in activating DDR, *Pf*SR1 might also contribute to parasite survival after exposure to artemisinin through the UPR machinery, which was recently implicated in the mechanisms of artemisinin resistance, as *Pf*SR1 is also interacting with components of this pathway (Bridgford et al., 2018).

RNA processing and splicing factors have been implicated as gatekeepers of genome integrity (Giaever et al., 2002). DNA damage has been shown to affect expression, localization and post-translational modifications of many splicing factors, which in turn are involved in regulation of the DDR at different levels (Dutertre et al., 2014; Shkreta and Chabot, 2015). For example phosphorylation of SRSF1, the human ortholog of *Pf*SR1, is regulated during the DDR, shifting the alternative splicing pattern of target genes to control cell survival (Leva et al., 2012). There are also indications that splicing factors might play a more direct role in DDR. For example, SRSF1 has been shown to play a protective role against DSBs by preventing R-loop formations (Li and Manley, 2005; Li et al., 2005). Interestingly, poly(ADP-ribose) polymerase (PARP), which acts as a molecular sensor for both single- and double-strand DNA breaks, is involved in recruitment of splicing to the site of DNA lesions for activation of a proper DDR (Mastrocola et al., 2013). In addition, the human splicing factor PFS (also known as SFPQ) directly interacts with RAD51 and may enhance its ability to repair DSBs by HR (Morozumi et al., 2009). Altogether, these studies provide evidence for a direct role of splicing factors in the DDR. To the best of our knowledge, we demonstrate for the first time that *Pf*SR1 is recruited to the site of damaged chromatin, where it interacts with γ-*Pf*H2A and colocalizes with *Pf*RAD51. Moreover, *Pf*SR1 expression is essential for *Pf*RAD51 accumulation in the nucleus and DNA repair. This association may suggest that *Pf*SR1 plays a functional role at the site of damaged DNA to preserve genome integrity after exposure to irradiation or artesunate. This conclusion does not exclude the possibility that *Pf*SR1 is also involved in other indirect regulatory pathways in the DDR machinery through regulation of gene expression.

Interestingly, the *Pf*SR1 interactome was enriched with several orthologs of proteins implicated in DDR, even in parasites that were not exposed to an exogenous source of DNA damage. These include: the mismatch repair protein MSH6, which localizes with γ-H2AX at sites of DNA damage and is involved in repairing DSBs by NHEJ (Bannister et al., 2004; Shahi et al., 2011; Smith et al., 2005), MCM4, MCM6 and DNA pol ε, which play an important role in maintaining genome integrity during replication (Bailis and Forsburg, 2004; Pedroza-García et al., 2017), and the DEAD-box DNA/RNA helicase 60, which was found to be important for clearance of R-loops to promote DNA repair (Pradhan et al., 2005; Yu et al., 2020) similar to the role of RNase H2A (Briggs et al., 2019; Williams et al., 2017). *Pf*SR1 interactions with these DDR proteins were observed during ring stage (before the parasite entered the S phase), thus indicating that in addition to its role in DNA repair after exposure to a source of damage, it could also be involved in additional mechanisms that protect genome integrity during IDC.

In addition to proteins involved in the DDR, the interactome of *Pf*SR1 contains many additional proteins involved in several processes of RNA metabolism, ranging from chromatin remodeling, transcription, splicing to translation. Previously, we provided evidence that in addition to its role as an alternative splicing (AS) factor, *Pf*SR1 binds to specific RNA motifs and regulates the steady-state RNA levels of a subset of RNA molecules (Eshar et al., 2015). Moreover, *Pf*SR1 localizes mainly to the nucleus at the early stages of the IDC, while in late stages it is located primarily in the cytoplasm of the parasites (Eshar et al., 2012). This dynamic localization indicates that *Pf*SR1 may have a multifaceted biological function both in the nucleus and the cytoplasm. In higher eukaryotes, SR proteins are recruited to transcriptionally active chromatin through the C-terminal domain of RNA pol II during transcription elongation, and the initial steps of RNA processing, such as pre-mRNA splicing, are coupled with transcription (Kornblihtt, 2007; Zhong et al., 2009). Coupling between transcription and mRNA processing depends on chromatin structure and its accessibility to splicing regulators (Schor et al., 2012). In agreement with these findings, we found that *Pf*SR1 is associated with Pol II as well as with transcription elongation factors and additional chromatin regulators, suggesting that transcription and splicing could be coupled in *P. falciparum* as well. In addition, direct and functional interactions of the SR proteins with specific chromatin modifications have been shown to be essential for proper cell cycle progression (Loomis et al., 2009). Our data show that downregulation of *Pf*SR1 causes a delay in parasite growth rate, however, further investigation is required to determine whether interactions of *Pf*SR1 with chromatin play a role in the cell cycle progression of malaria parasites. Besides its interaction with components of the transcriptional machinery, *Pf*SR1 also interacts with another SR protein (PF3D7_0503300), which has significant similarity with the mammalian SRSF2 and SRSF12. Interestingly, SRSF2 was shown to be restricted to the nucleus where it associates with DNA (Sapra et al., 2009) and recently was shown to play a role in regulating transcriptional activation by releasing paused Pol II on gene promoters (Ji et al., 2013). It will be interesting to further investigate the possibility that *Pf*SR1 is involved in transcriptional regulation.

SR proteins have also shown to be involved in mRNA export as well as in regulation of cytoplasmic processes, such as mRNA decay and translation. In the late stages of the IDC, *Pf*SR1 was found to be localized mainly to the cytoplasm (Eshar et al., 2012), but its cytoplasmic function was never investigated. Interestingly, we found that most of the *Pf*SR1 interactions with other proteins occur at 20–24 h post infection. During this first phase of the IDC, *Pf*SR1 interacts with proteins involved in ribosome biogenesis and translation. This could indicate that *Pf*SR1 shuttling to the cytoplasm has already begun at that phase, and that *Pf*SR1 might also be involved in the translation process. Cytoplasmic SR proteins have been found to interact with actively translating ribosomes and are capable of stimulating protein synthesis by promoting mRNA entrance to polysomes (Sanford et al., 2005, 2004), stimulating mTOR, and/or enhancing phosphorylation of 4E-BP1 (Karni et al., 2008; Michlewski et al., 2008). The interactions of *Pf*SR1 with polyadenylated RNA-binding protein, the translation initiation factor EIF-2B and other ribosomal proteins may indicate that, similar to SRSF1, it could contribute to translational enhancement in the cytoplasm. Interestingly, 36% of the binding partners of SRSF1 are ribosomal proteins, and its specific interaction with RPL5 induces cellular senescence (Fregoso et al., 2013), which is essential for DNA damage repair. Altogether, our data point towards a novel role of *Pf*SR1 in protecting *P. falciparum* parasites from different sources of DNA damage and opens new avenues for investigation into the mechanisms involved.

## MATERIALS AND METHODS

### Parasite culture and parasitemia counts

All parasites used were derivatives of the NF54 parasite line and were cultivated at 5% hematocrit in RPMI 1640 medium, 0.5% Albumax II (Invitrogen), 0.25% sodium bicarbonate and 0.1 mg/ml gentamicin (complete culture medium). Parasites were incubated at 37°C in an atmosphere of 5% oxygen, 5% carbon dioxide and 90% nitrogen. Parasite cultures were synchronized using Percoll/sorbitol gradient centrifugation as previously described (Aley et al., 1984; Calderwood et al., 2003). Briefly, infected red blood cells (RBCs) were layered on a step gradient of 40%/70% Percoll containing 6% sorbitol. The gradients were then centrifuged at 12,000 ***g*** for 20 min (F15-6×110y fixed angle rotor; Thermo Fisher Scientific) at room temperature. Highly synchronized late stage parasites were recovered from the 40%/70% interface, washed twice with complete culture medium and placed back in culture. The level of parasitemia was calculated by flow cytometry. For flow cytometry, aliquots of 50 µl parasite cultures were washed in PBS and incubated 30 min with 1:1000 SYBR Green I DNA stain (Life Technologies). The fluorescence profiles of infected erythrocytes were measured on CytoFLEX (Beckman Coulter) and analyzed by the CytExpert software.

### Plasmid construction

In order to express *Pf*SR1 fused to a Halo tag, a Halo tag sequence was amplified using the primers nHalo-F and nHaloR (5′-GGCGACTAGTCCATGGCAGAAATCG-3′ and 5′-CCGCGAGCTCTGAATTCGGAAGCGATC-3′) and cloned into the expression vector pHBIRH (Eshar et al., 2012) using SpeI/SacI (essentially replacing *Renilla* luciferase and the hrp2 3′ and introducing an AsiSI restriction site) to generate the pHBInHalo vector. Then *Pfsr1* (PF3D7_0517300) was amplified together with 1000 bp of its 3′ UTR using the primers n*Pf*sr1F and n*Pfsr*1-UTR-R (5′-CCAGCGATCGCAATGGTTATACGTGAAAGT-3′ and 5′-CCGGAGCTCATATCTATATTTTTGTAAA-3′) and cloned into pHBInHalo using *AsisI–SacI* to generate pHBInHalo*Pf*SR1UTR. To create the *Pf*SR1-*glmS* transgenic line, we used a modified CRISPR/Cas9 previously described (Ghorbal et al., 2014). The Cas9 nuclease was expressed with pUF1-Cas9. The pL6-*Pf*SR1-HA-*glmS* plasmid, was constructed by amplifying *Pf*SR1 homology arm from the 5′ of its open reading frame (ORF) using primers 5′-AGGTGAATGTGGTCATGCAG-3′ and 5′-ATGTCTTCTTTTATGGGACGATGATG-3′. The endogenous *Pf*SR1 3′ UTR was amplified using the primers: 5′-CGTCCCATAAAAGAAGACATTAGA-3′ and 5′-ATGCTTAAGCAGTGCGAGGCTCTATTATGTG-3′. Then, both fragments were cloned into pL7-*Pf*SAC1-3HA-*glmS*-DHFR (Thériault and Richard, 2017) using Not1/Sma1 and Nhel/Afll1, respectively, to generate the pL6-*Pf*SR1-3HA-*glmS*-DHFR. For cloning the sgRNA, we used the In-Fusion HD Cloning Kit (Clontech), with the 20-bp guide RNA (5′-CCAACTCAAGATCATCATCA-3′) surrounded by the 15 bp necessary for In Fusion cloning. The final pL6-*Pf*SR1-3HA-*glmS*-DHFR constructs were made by replacement of the BtgZI-adaptor with the guide RNA sequence. All PCR amplifications were done with high-fidelity Q5 Taq polymerase (New England Biolab).

### Parasite transfection and selection

Parasites were transfected as described previously (Deitsch et al., 2001). Briefly, 0.2 cm electroporation cuvettes were loaded with 0.175 ml of erythrocytes and 50 µg of plasmid DNA in an incomplete cytomix solution. Stable transfectants carrying plasmids with an hDHFR-selectable marker were selected on 4 nM WR99210 and those carrying yDHODH were selected on 1.5 µM DSM1. Stable transfectants carrying plasmids with BSD-selectable marker were initially selected on 2 μg/ml blasticidin-S (Invitrogen). In order to obtain parasites carrying large plasmid copy numbers, these cultures were then subjected to elevated concentrations of 6–10 µg/ml blasticidin-S, depending on experimental design.

### HaloLinK pulldown assay

The Halo pulldown assay was performed on extracts made from 400–500 ml of tightly synchronized parasite cultures of early (20 hpi) and late (36 hpi) stages. Infected (i)RBCs were saponin lysed (50 μl per 100 ml culture), washed twice in PBS and the parasite pellets were stored at −80°C for at least 30 min. Parasite pellets were then thawed in 200 μl lysis buffer (50 mM Tris-HCl pH 7.5, 150 mM NaCl, 1% Triton X-100 and 0.1% sodium deoxycholate) with protease inhibitor cocktail (Promega, cat # G6521), mixed well by pipetting and incubated on ice for 15–30 min. Pellets were then centrifuged at 14,000 ***g*** for 5 min at 4°C and the supernatant was transferred into a fresh new tube added with TBS to the final volume of 1 ml [80 μl of this starting material (SM) was kept for western blotting]. Pull down of Halo–*Pf*SR1-interacting proteins was performed using the HaloTag^®^ Protein Purification System (Promega, cat #G1913) according to the manufacturer's guidelines. Briefly, HaloLink resin was washed 3–5 times with 800 μl wash buffer (90 μl of 10% NP40 in 18 ml TBS) and the sample was added to the HaloLink resin and incubated in 4°C overnight while rotating. The following day the sample was centrifuged and the resin was washed 3 times in wash buffer while the flow through (FT) was kept for quality control by western blotting. To recover the purified proteins of interest (without the resin bound tag), elution was performed by re-suspending the resin in 50 μl SDS solution buffer and incubating for 30 min at 1400 rpm at 55°C. The starting materials, flow through fractions and eluates were all checked by western blotting for the presence of the Halo tag. To verify specific enrichment in the Halo–*Pf*SR1 pulldown, the purified proteins were loaded on an SDS-PAGE and silver stained for protein visualization (Pierce Silver Stain Kit, Thermo Scientific). Coomassie-stained SDS-PAGE samples were sent for mass spectrometry analysis.

### LC-MS/MS analysis and database analysis

The proteins obtained by HaloLink pulldown were separated on a 4–20% acrylamide gel (BioRad) and stained with NOVEX Colloidal Blue Staining Kit (Invitrogen). The samples were digested by trypsin, analyzed by LC-MS/MS on Q Exactive plus (Thermo) and identified by Discoverer software version 1.4 against the *Plasmodium* NCBI-NR database and against decoy databases in order to determine the false discovery rate (FDR) using the sequest and mascot search engines. Semi quantification was undertaken by calculating the peak area of each peptide. The area of the protein is the average of the three most intense peptides from each protein. The results were filtered for proteins identified with at least two peptides with 1% FDR. We considered specific *Pf*SR1 enrichment as proteins that were enriched by either more than 8-fold or found only in the Halo–*Pf*SR1 pulldown fraction and not in proteins extracted from parasites transfected with the mock plasmid, in at least two replicates.

### Southern blotting

Analysis of the integrated construct was performed using Southern blots and diagnostic PCR crossing the integration sites (using primers P1F, 5′-ACGTGAAAGTGTATCGAGAA-3′; P2R 5′-AGTGCGAGGCTCTATTATGTG-3′; and P3R, 5′-ATGCCTTTCTCCTCCTGGAC-3′) followed by sequencing. Southern blots were performed according to established protocols (Dahan-Pasternak et al., 2013; Sambrook et al., 1989). Briefly, genomic DNA isolated from recombinant parasites was digested to completion with the restriction enzymes SpeI and NheI and subjected to gel electrophoresis using 1% agarose gel in Tris/Borate/EDTA (TBE). The DNA was transferred to a high-bond nitrocellulose membrane by capillary action after alkaline denaturation. DNA detection was performed using DIG High Prime DNA Labeling and Detection starter kit (Roche). The *glmS* sequence amplified from pL6-HA-*glmS* using 5′-GATTATGCCTAATCTTGTTCTT-3′ and 5′-TAGCATTTTTCTTCCTCCTAAGAT-3′ was Dig labeled and used as a probe.

### Immunofluorescence assay

Immunofluorescence assays (IFAs) was performed as described previously with a few modifications (Dahan-Pasternak et al., 2013). Briefly, 1 ml of parasite culture at 5% parasitemia was washed with PBS and re-suspended in a fresh fixative solution [4% paraformaldehyde (EMS) and 0.0075% glutaraldehyde (EMS) in PBS]. Fixed parasites were treated with 0.1% Triton-X 100 (Sigma) in PBS, then blocked with 3% BSA (Sigma) in PBS. Cells were then incubated with the following primary antibodies, used at the indicated dilutions, mouse anti-HA (Roche, 1:300), rabbit-anti-γ-H2A.X (Cell Signaling, cat # 9718S, 1:300), rabbit anti-Rad51 1:100 (Gene Tex cat # GTX100469), incubated for 1.5 h and washed three times in PBS. Samples were incubated with Alexa Fluor 488 goat anti-mouse-IgG (Life Technologies, 1:500) and Alexa Fluor 594 goat anti-rabbit-IgG (Life Technologies, 1:500) antibodies. Samples were washed and laid on ‘PTFE’ printed slides (EMS) and mounted in ProLong Gold antifade reagent with DAPI (Molecular Probes). Fluorescent images were obtained using a Plan Apo λ 100× oil NA 1.5 WD 130 µm lens on a Nikon Eclipse Ti-E microscope equipped with a CoolSNAP Myo CCD camera. Images were processed using the NIS-Elements AR (4.40 version) software.

### Western blotting

To collect parasite proteins, iRBCs were lysed with saponin, then the parasites were washed twice with PBS and lysed in Laemmli sample buffer (Sigma). Equal amount of denatured protein samples were subjected to SDS-PAGE (4–20% gradient gels, Bio-Rad) and electroblotted onto a nitrocellulose membrane. Immunodetection was carried out using rabbit polyclonal anti-Halo antibody (Promega; 1:1000), mouse anti-HA antibody (Roche; 1:1000), rabbit anti-γH2A.X antibody (Cell Signaling cat # 9718S, 1:1000), rabbit anti-Rad51 (Gene Tex cat # GTX100469, 1:100) and rabbit polyclonal anti-aldolase (1:2000) (Dahan-Pasternak et al., 2013) antibodies. The secondary antibodies used were goat anti-rabbit and goat anti-mouse-IgG antibodies conjugated to horseradish peroxidase (HRP; Jackson ImmunoResearch Laboratories, 1:10,000). The immunoblots were developed in EZ/ECL solution (Israel Biological Industries).

### X-ray irradiation of parasites

Parasites were irradiated using a PXi precision X-ray irradiator set at 225 kV, 13.28 mA. Prior to irradiation parasitemia was quantified by flow cytometry. A starting parasitemia of 0.5% ring stage parasites in the control and the knockdown populations were both exposed to 10–60-Gy (1000-6000 rad) X-ray irradiation. After irradiation parasites were either collected immediately or put back in culture and medium was replaced daily. Population recovery was measured by flow cytometry daily. To detect the increase in damage following irradiation, we collected parasites 15 min after irradiation and measured the levels of phosphorylated *Pf*H2A by western blotting as described above.

### *In situ* DNA fragmentation assay

Tightly synchronized ring stages parasites (NF54 and *Pf*SR1) were fixed for 30 min in freshly prepared fixative (4% paraformaldehyde and 0.005% glutaraldehyde). After fixation cells were rinsed three times with PBS and incubated with a permeabilization solution (0.1% Triton X-100 in PBS) for 10 min on ice. The cells were washed twice with PBS, and once with wash buffer supplied with the TUNEL Assay Kit-BrdU Red (Abcam cat # ab6610). The TUNEL assay was performed as per manufacturer's guidelines. Briefly, following washing, 50 μl of TUNEL reaction mixture (DNA-labeling solution) was added to each sample. The cells were incubated for 60 min at 37°C with intermittent shaking. Cells were then washed three times with a rinse buffer (5 min each time) and re-suspended in 100 μl of antibody solution for 30 min at room temperature. Cells were then washed three times with PBS and mounted using Invitrogen™ Molecular Probes™ ProLong™ Gold Antifade reagent with DAPI, and imaged using a fluorescence microscope as described above.

### Differential salt fractionation of nuclei to analyze chromatin association of *Pf*SR1

*Pf*SR1 association with chromatin was measured by isolating nuclei and separation of the high salt (strong chromatin association) and low salt (weak chromatin association) fractions before and after X-ray irradiation (6000 rad) of *Pf*SR1-*glmS* parasites. In brief, 100 ml of parasite culture (∼10% parasitemia) was subjected to X-ray irradiation. Immediately following irradiation, parasites were released by saponin lysis and washed twice with 1× PBS-containing protease inhibitor cocktail. Equal number of parasites of each treatment were incubated with nuclei isolation buffer [10 mM Tris-HCl pH 7.5, 2.5 mM MgCl_2_, 14 mM 2-mercaptoethanol (added fresh), 0.5% Igepal/Nonidet P-40] containing protease inhibitor cocktail. The cells were kept on ice for 10–30 min with intermittent shaking. Following incubation, nuclei were pelleted down by centrifugation at 2500 ***g*** for 5 min at 4°C. The nuclei were washed three times with nuclei isolation buffer and re-suspended by gentle pipetting in two volumes of ice-cold buffer A (10 mM Tris-HCl, pH 8.0, 100 mM NaCl, 1 mM EDTA, 0.5 mM EGTA supplemented with protease inhibitor cocktail) and incubated for 10 min. The soluble nuclear proteins (supernatant) were separated from chromatin-bound proteins (pellet) by centrifugation at 2500 ***g*** for 5 min at 4°C. The pellet was washed twice with buffer A and either re-suspended directly in 2× Laemmli buffer or re-suspended in buffer B (50 mM Tris-HCl pH 7.5, 1 M NaCl, 0.05% NP40) and subjected to mild sonication in a water bath sonicator (10 s pulse followed by 50 s rest at 50% amplitude). All the fractions were centrifuged at 16,000 ***g*** for 10 min at 4°C before being subjected to western blotting. Anti-γ-*Pf*H2A levels were used to demonstrate that damage had occurred after irradiation and determining the equal fractionation efficiency.

### Co-immunoprecipitation

Immunoprecipitation was performed according to Stewart et al. (2003) with a slight modification. In brief, 200 ml of parasite cultures (∼10% parasitemia) were saponin lysed, and washed with PBS-containing protease inhibitors. Subsequently, the parasite pellet was dissolved in chilled lysis buffer containing 50 mM Tris-HCl pH 7.5, 150 mM NaCl, 1 mM EDTA, 0.1% SDS and 1% NP40 supplemented with protease inhibitors (Roche) and sonicated for 4 cycles of 10–15 s at 45% output using a Hielscher UP200S sonicator. The sonicated pellet was incubated for 30 min on ice. The lysate was purified by a few rounds of centrifugations at 10,000 ***g*** for 10 min and incubated with a primary anti-γ-H2A.X (Abcam cat # Ab2893, 1:300) antibody for 10–12 h at 4°C with continuous swirling. The supernatant was further incubated for 12–14 h with Protein A/G agarose beads (Pierce) at 4°C, and then the beads were pelleted by centrifugation at 4°C. Beads were then washed with ice-chilled washing buffer. Immunoprecipitated proteins were eluted with SDS Laemmli buffer and used for detection by SDS-PAGE and western blot analysis.

### DNA repair assay

Tightly synchronized ring stage parasites were exposed to 10 Gy X-ray irradiation using a PXi irradiator as described above (Goyal et al., 2021). Immediately following irradiation, parasites were put back to culture to allow them to repair the damaged DNA. Protein was extracted 15 min after irradiation (0 h), as well as from parasites collected at 3 h and 6 h after irradiation. Proteins extracted from untreated iRBCs were used as control. Western blot analysis was used to follow the changes in γ-*Pf*H2A compared with the housekeeping control gene aldolase in each treatment. These western blots were subjected to densitometry analysis to calculate the ratio between γ-*Pf*H2A levels and aldolase.

## Supplementary Material

Supplementary information

Reviewer comments
