# Supplementary material for: An SR protein is essential for activating DNA repair in malaria parasites
Source: J Cell Sci. 2021 Aug 17;134(16):jcs258572. doi: 10.1242/jcs.258572 (PMC8435287; doi:10.1242/jcs.258572)
Supplement: Supplementary information [file joces-134-258572-s1.pdf]

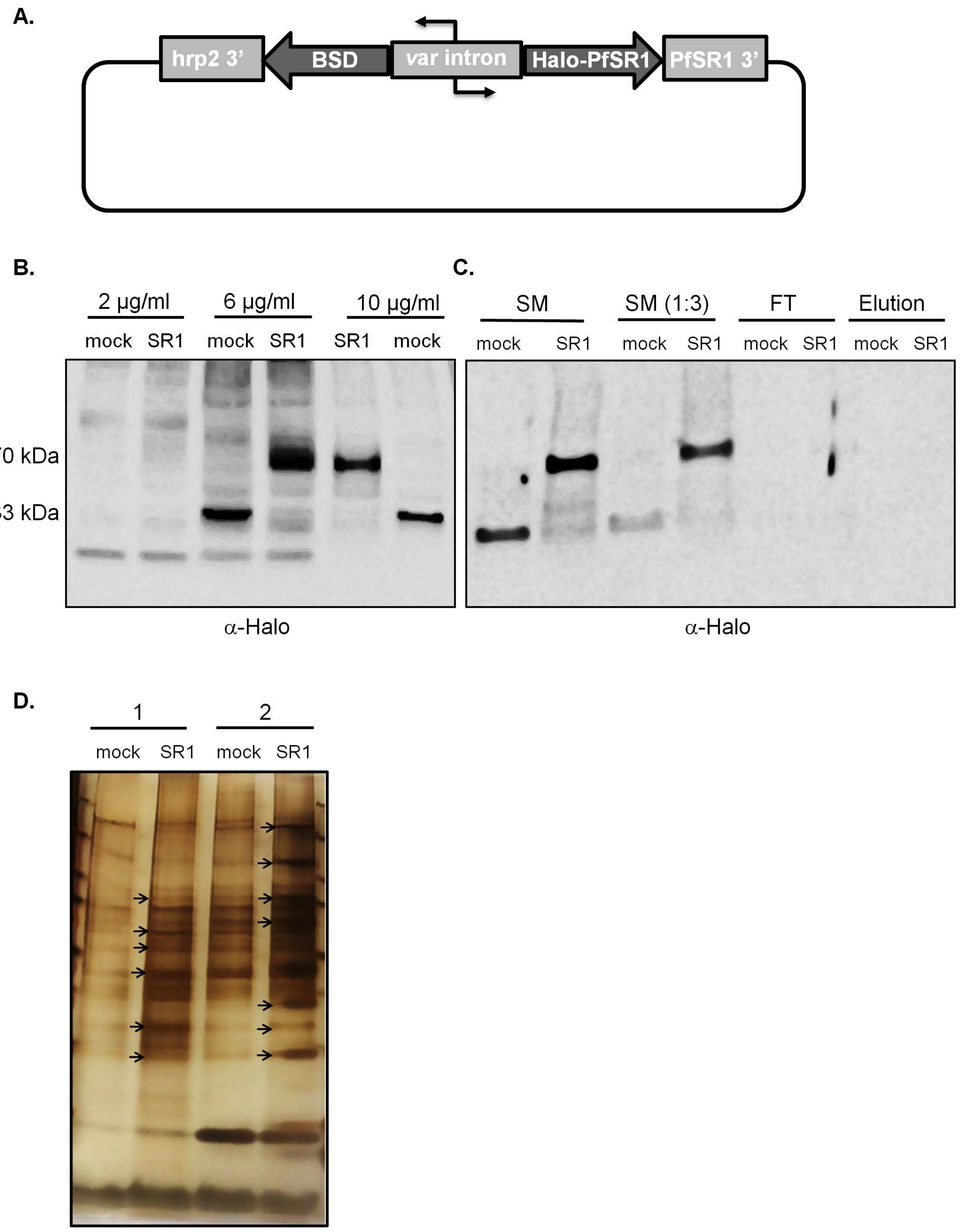

Figure S1. Identification of *Pf*SR1 interacting proteins using Halo-tag pull down. (A). Scheme of the plasmid used to express N' terminal fusion of the Halo-tag with *Pf*SR1. The use of *bsd* resistant gene as selectable marker allows gradual over expression of episomal Halo-*Pf*SR1. **(B).** Gradual over expression of Halo-*Pf*SR1 (SR1) and the mock plasmid (mock) that expresses the Halo tag only, on increasing concentrations of blasticidin S. Western Blot analysis using anti Halo antibody, indicating that protein expression can be detected on 6 and 10 µg/ml blasicidin (Halo tag only, ~33 kDa and Halo-*Pf*SR1, ~70 kDa). **(C).** Western blot analysis of the Halo pull down assay, showing expression of Halo-*Pf*SR1 (SR1) and the Halo-tag only (mock). SM, starting material; SM (1:3), starting material diluted in 3 volumes of TBS buffer; FT, flow through; Elution, protein eluted from beads. The absence of Halo signal in the elution fraction indicates that all the proteins expressing Halo (tagged *Pf*SR1 or the tag alone) were attached to the beads, while co-IP proteins came in the elution. **(D).** Silver stain analysis of the protein which were pulled down in the eluted fraction in C. Two biological replicates of the proteins which were pulled down with either the Halo-*Pf*SR1 (SR1) the Halo-tag only (mock). Proteins which were specifically enriched in the Halo-*Pf*SR1 are indicated by arrows.

Journal of Cell Science • Supplementary information

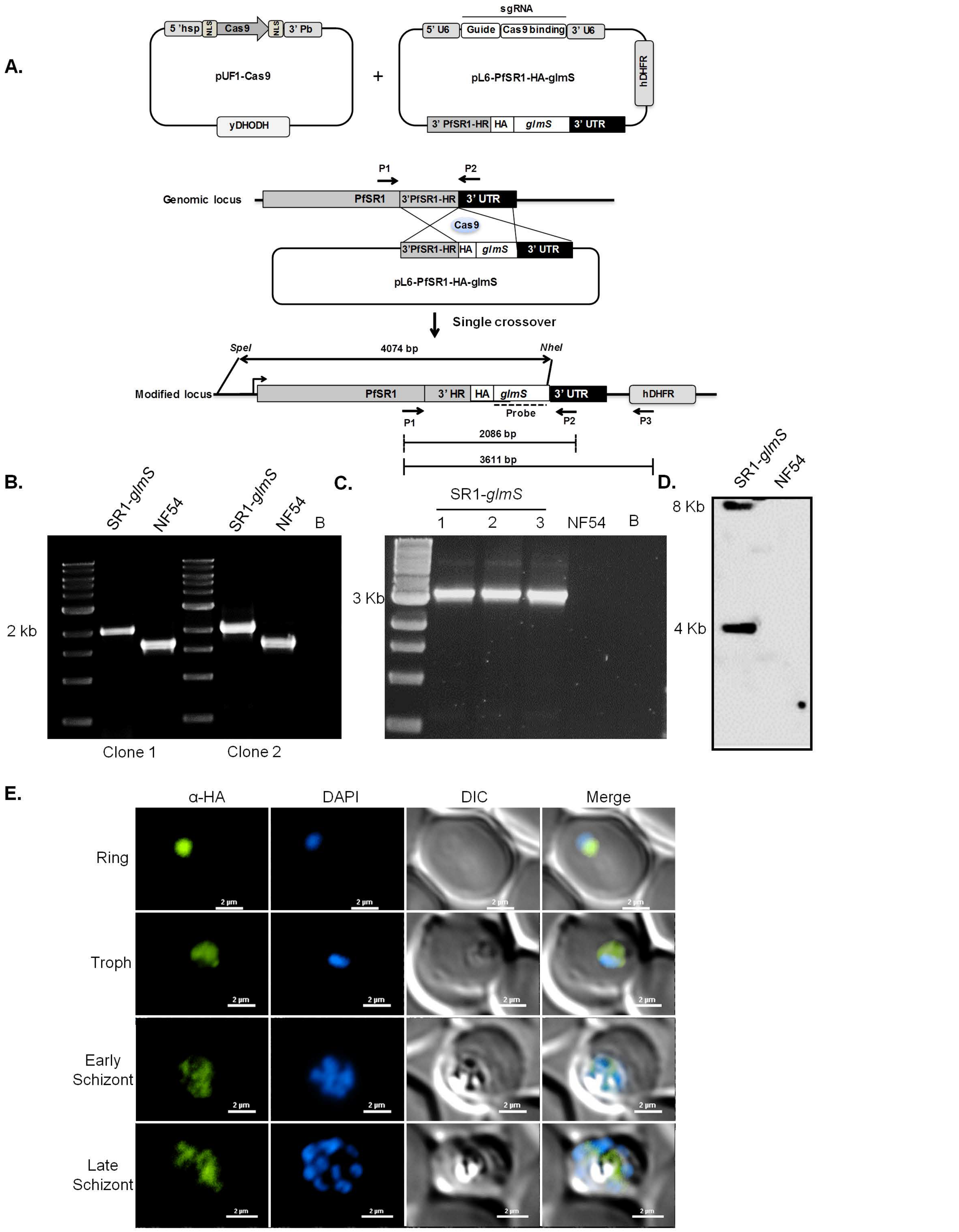

Figure S2. Generation of a transgenic parasite line in which *Pf*SR1 could be knocked-down using the CRISPR/ cas9 system. (A). Scheme of the plasmids used to endogenously tag *Pf*SR1 with HA tag and *glmS* ribozyme by 3' replacement using CRISPR/cas9 system. Left: the expression vector expressing the cas9 nuclease using yDHODH as selectable marker. Right: the plasmid containing the sgRNA, 3' *Pf*SR1 homology region fused to HA epitope, *glmS* ribozyme and the endogenous *Pf*SR1 3' UTR. These plasmids were designed to integrate by double cross over recombination into the genomic locus of *Pf*SR1 (middle). However, we got a single cross over integration as seen in the map of genomic integration presented below. The integration map is marked for primers used to detect integration by PCR, restriction sites, and the probe used for Southern blot. **(B-C)**. PCR analysis of the transgenic *Pf*SR1-*glmS* parasites by using primers P1 & P2 to amplify across integration events of different amplicon sizes (B), or positive amplification using primers P1 & P3 (C) in 3 different clones and wt NF54 population. B, blank sample with no DNA template. **(D).** Southern blot analysis using a probe designated to the *glmS* sequence demonstrating integration by 3' replacement (4Kb) and a full size linearized plasmid corresponding with concatameric integration (8Kb). **(E).** Immuno fluorescence assay demonstrating expression of HA-tagged endogenous *Pf*SR1 during different stages of IDC. Scale bar, 2µm.

Journal of Cell Science • Supplementary information

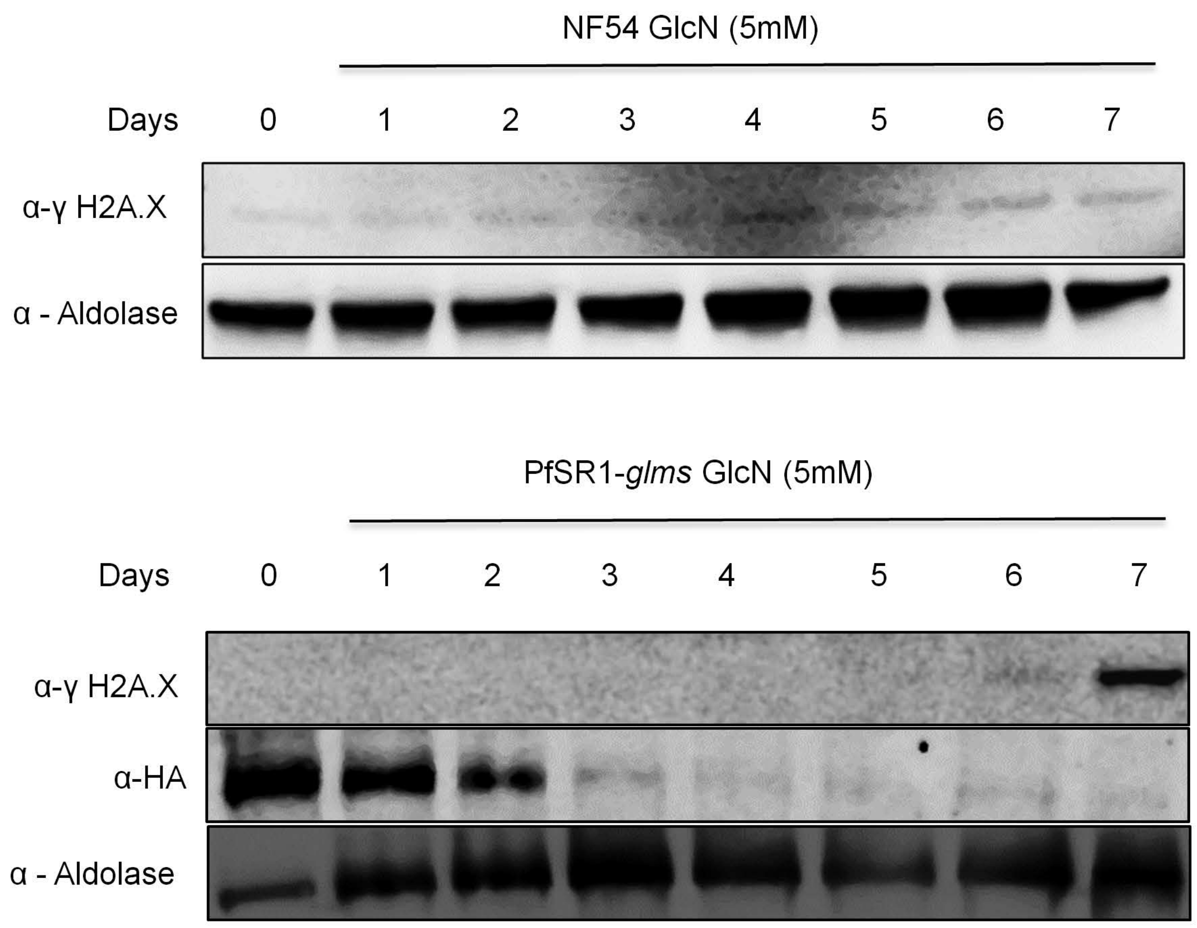

Figure S3. Western blot analysis of NF54 parasite line growing on GlcN over time, indicating that γ-*Pf*H2A is detected at basal level after one week (upper panel), while it accumulates when *Pf*SR1 is downregulated (lower panel).

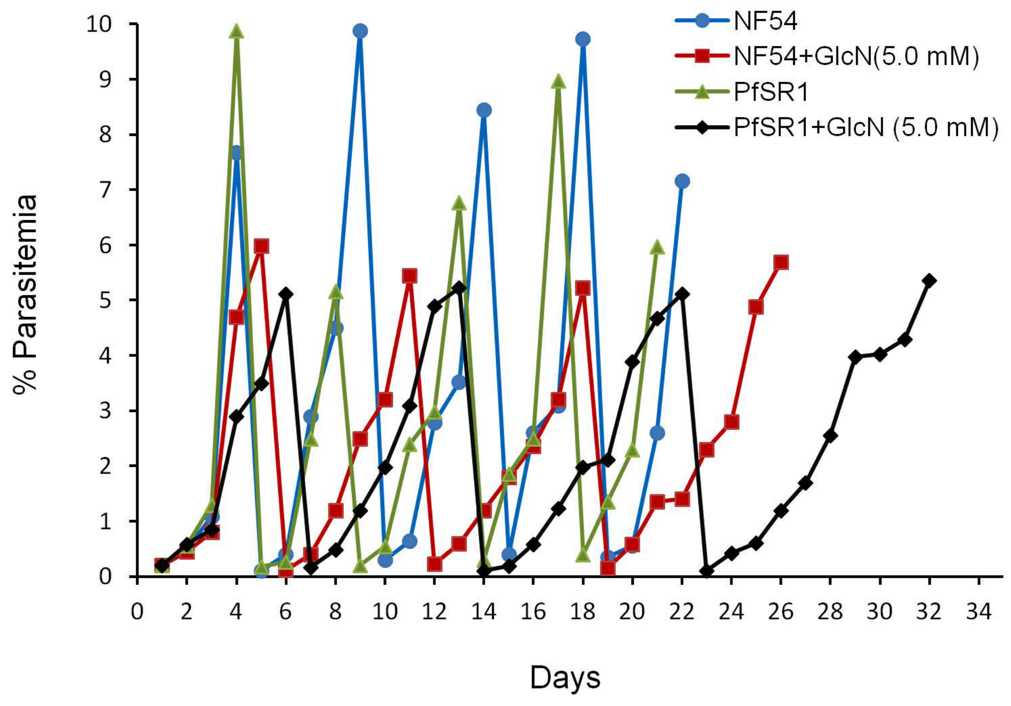

Figure S4. *Pf*SR1 down regulation leads to a slower growth rate over time. Long term growth curves of NF54 and *Pf*SR1-*glmS* parasites grown either on regular media or media supplemented with 5 mM GlcN (5.0 GlcN). Media was replaced every day with fresh media with or without glucosamine respectively. Cultures that reached 5% parasitemia and above were cut down to avoid over-parasitemia over the course of experiment as previously described (Eshar et al., 2012).

Journal of Cell Science • Supplementary information

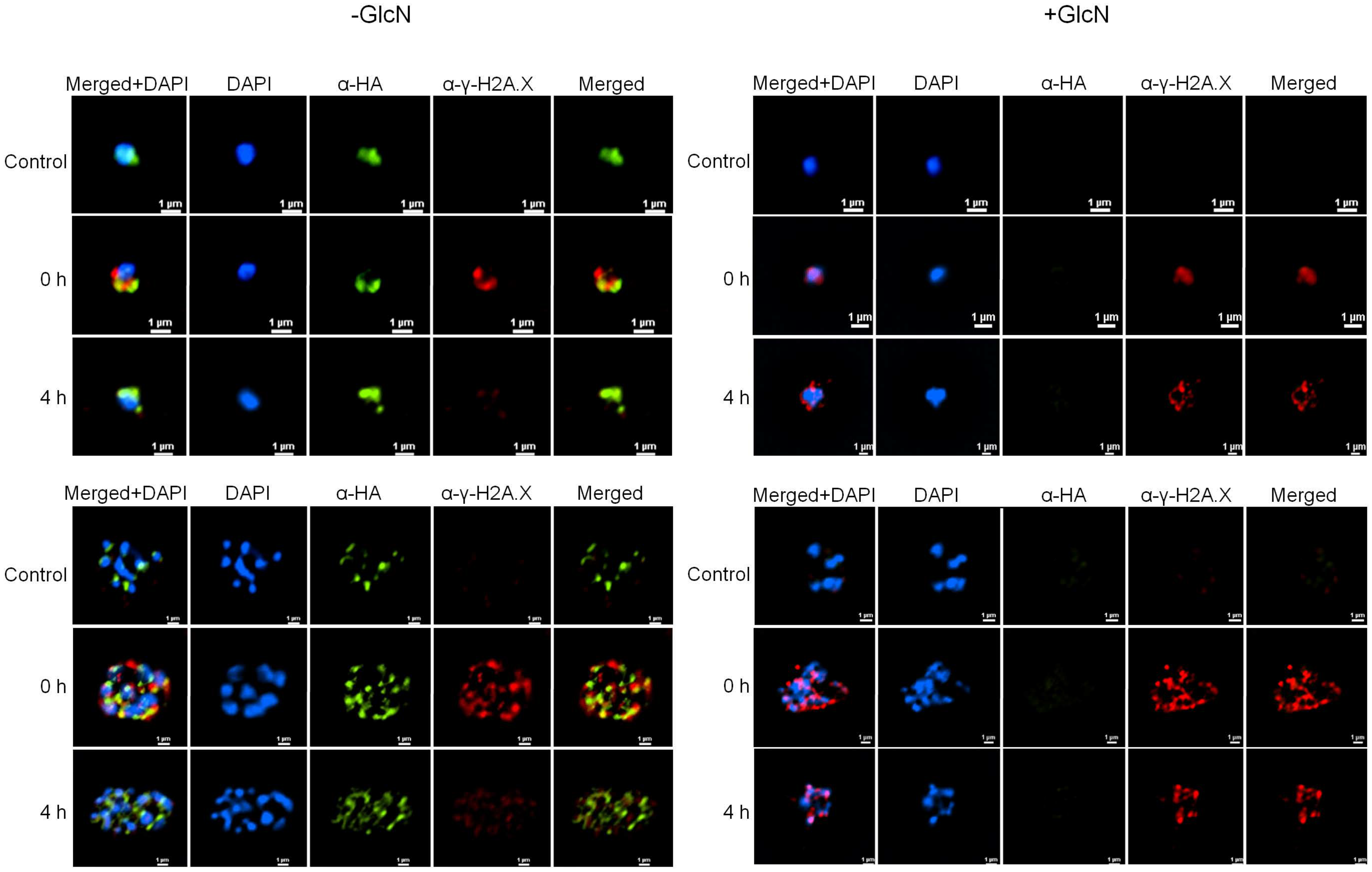

Figure S5. Dynamics of *Pf*H2A phosphorylation in *P. falciparum* nucleus following X-ray induced DNA damage in the presence or absence of *Pf*SR1 expression. Immunofluorescence imaging of γ-*Pf*H2A (red) and *Pf*SR1 (green) in the nucleus of early (upper panels) and late stages (lower panels) *Pf*SR1-*glmS* parasites grown 72h either on regular media (-GlcN, left panels) or media supplemented with 5mM GlcN (+ GlcN, right panels) to knockdown *Pf*SR1 expression. Parasite were exposed to X-ray irradiation (3000 rad) and the association between *Pf*SR1 and the γ-*Pf*H2A foci formation was imaged before irradiation (Control), 15 minutes after exposure (0h), and 4 hour post (4h) X-ray irradiation. DNA is stained with DAPI, scale bar 1 µm.

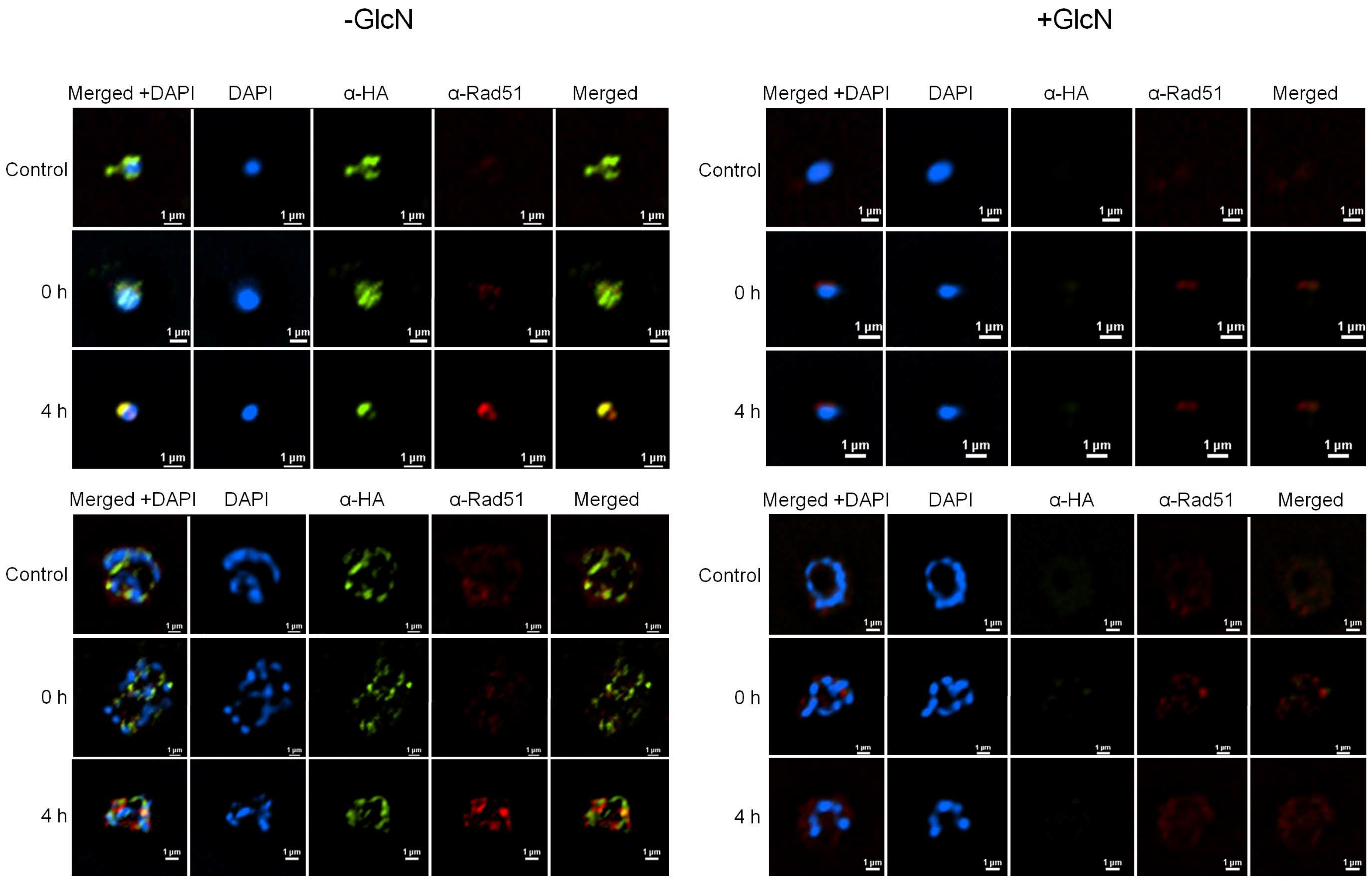

Figure S6. Cellular dynamics of *Pf*Rad51 following X-ray induced DNA damage in the presence or absence of *Pf*SR1 expression. Immunofluorescence imaging of *Pf*Rad51 (red) and *Pf*SR1 (green) in the nucleus of early (upper panels) and late stages (lower panels) *Pf*SR1-*glmS* parasites grown 72h either on regular media (-GlcN, left panels) or media supplemented with 5mM GlcN (+ GlcN, right panels) to knockdown *Pf*SR1 expression. Parasite were exposed to X-ray irradiation (3000 rad) and the association between *Pf*SR1 and *Pf*Rad51 accumulation in the nucleus was imaged before irradiation (Control), 15 minutes after exposure (0h), and 4 hour post (4h) X-ray irradiation. DNA is stained with DAPI, scale bar 1 µm.

Journal of Cell Science • Supplementary information

Table S1. List of *Pf*SR1 interacting proteins identified by Halo-tag pull down followed by LC-MS/MS.

Click here to download Table S1

Reference

Eshar, S., Allemand, E., Sebag, A., Glaser, F., Muchardt, C., Mandel-Gutfreund, Y., Karni, R. and Dzikowski, R. (2012). A novel Plasmodium falciparum SR protein is an alternative splicing factor required for the parasites' proliferation in human erythrocytes. *Nucleic Acids Res* **40**, 9903-16.

Journal of Cell Science • Supplementary information
