# Supplementary material for: An SR protein is essential for activating DNA repair in malaria parasites
Source: J Cell Sci. 2021 Aug 17;134(16):jcs258572. doi: 10.1242/jcs.258572 (PMC8435287; doi:10.1242/jcs.258572)
Supplement: Reviewer comments [file joces-134-258572_review_history.pdf]

An SR protein is essential for activating DNA repair in malaria parasites

Manish Goyal, Brajesh Kumar Singh, Karina Simantov, Yotam Kaufman, Shiri Eshar and Ron Dzikowski
DOI: 10.1242/jcs.258572

Editor: Maria Carmo-Fonseca

Review timeline

Original submission:	22 February 2021
Editorial decision:	16 March 2021
First revision received:	16 June 2021
Accepted:	14 July 2021

Original submission

First decision letter

MS ID#: JOCES/2021/258572

MS TITLE: An SR protein is essential for activating DNA repair in malaria parasites

AUTHORS: Brajesh Kumar Singh, Manish Goyal, Karina Simantov, Yotam Kaufman, Shiri Eshar, and Ron Dzikowski
ARTICLE TYPE: Research Article

We have now reached a decision on the above manuscript.

To see the reviewers' reports and a copy of this decision letter, please go to: https://submit-jcs.biologists.org and click on the 'Manuscripts with Decisions' queue in the Author Area. (Corresponding author only has access to reviews.)

As you will see, the reviewers raise a number of substantial criticisms that prevent me from accepting the paper at this stage. They suggest, however, that a revised version might prove acceptable, if you can address their concerns. If you think that you can deal satisfactorily with the criticisms on revision, I would be pleased to see a revised manuscript. We would then return it to the reviewers.

We are aware that you may be experiencing disruption to the normal running of your lab that makes experimental revisions challenging. If it would be helpful, we encourage you to contact us to discuss your revision in greater detail. Please send us a point-by-point response indicating where you are able to address concerns raised (either experimentally or by changes to the text) and where you will not be able to do so within the normal timeframe of a revision. We will then provide further guidance. Please also note that we are happy to extend revision timeframes as necessary.

Please ensure that you clearly highlight all changes made in the revised manuscript. Please avoid using 'Tracked changes' in Word files as these are lost in PDF conversion.

I should be grateful if you would also provide a point-by-point response detailing how you have dealt with the points raised by the reviewers in the 'Response to Reviewers' box. Please attend to

© 2021. Published by The Company of Biologists under the terms of the Creative Commons Attribution License (https://creativecommons.org/licenses/by/4.0/).

all of the reviewers' comments. If you do not agree with any of their criticisms or suggestions please explain clearly why this is so.

Reviewer 1

Advance summary and potential significance to field

Singh et al present and interesting, scientifically sound paper on the role for a splicing factor in the DNA damage response of the malaria parasite, P falciparum.
They have multiple lines of evidence to support a role of the PfSR1 in DNA repair and show that the PfSR1 interacts directly with chromatin, supporting a more direct role in DNA repair than a role of controlling the abundance of DNA repair proteins.

Comments for the author

I recommend revising the paper mostly in the presentation of the data included.
The paper would be improved by re-focusing the intro and conclusion on DNA repair and providing a hypothesis on the potential roles that SR1 may play in the parasite DDR within the context of what is known in higher eukaryotes. They reference review papers on the topic but do not explicitly provide any ideas on mechanism based on the literature and/or the data they present.

Parts of the abstract should be re-phrased. They state that PfSR1 is "required for activating DNA machinery" but do not have evidence of this, merely that the knockdown is delayed in its ability to repair DNA damage. They also state that "loss of PfSR1 does not impair parasite viability" yet in figure 1D and supplement 4 they demonstrate a slow growth phenotype which is likely multifactorial. In addition, they focus their discussion on replicative stress, yet the late stage interactome indicates less impact of SR1 at this stage when the parasite is actively replicating its DNA and the growth delay appears to be minimal (though hard to tell from supplementary fig 4) and is likely multifactorial.

I would recommend restructuring Figure 1. The small number of proteins at the ring stage make the late stage pie chart minimally informative. As this is a DNA repair focused paper, a table of the DNA repair proteins identified is much more meaningful and those proteins should be part of their discussion. In addition, the gene annotations in the interaction networks are outdated and should be updated to match those used in the supplementary table and current databases.
They present data that Rad51 and PfSR1 are both recruited to sites of DNA damage post irradiation. If they demonstrated that Rad51 is not recruited to sites of damage post irradiation in the knockdown, then that would support the hypothesis that PfSR1 is required to activate the DNA repair machinery thus providing insight into the mechanism for the persistent DNA damage in the PfSR1 knockdown.
It appears they are using DHA merely as a mutagen, albeit a potentially clinically relevant mutagen, and are not proposing that PfSR1 contributes to DHA mechanism of action or resistance. This should be clarified, especially in light of the UPS proteins identified in the interactome studies. The UPS has been identified as key to ART resistance and is equally or more likely to impact ART survival and this should at least be discussed.

Minor comments:
- In the intro and discussion they mention only a subset of DNA repair pathways present in the parasite, ie only MMR for non DSB repair. They should be comprehensive in their discussion or state they are only focusing on DNA DSBs.
- Supplemental fig 4 would be more informative transformed into a growth line

Reviewer 2

Advance summary and potential significance to field

In this manuscript the authors investigated the role of PfSR1 in the DNA damage repair (DDR) machinery in the human malaria parasites, Plasmodium falciparum. Even though PfSR1 was

© 2021. Published by The Company of Biologists under the terms of the Creative Commons Attribution License (https://creativecommons.org/licenses/by/4.0/).

originally linked with RNA splicing, which was demonstrated by the same group, here it was shown the PfSR1 protein interacts directly with multiple partners some of which play roles in DDR. Based on that here it as showen that glmS ribozyme-
mediated knock down of PfSR1leads to multiple DDR-linked phenotypes. These include, slow-to-no recovery of parasites after X-ray irradiation, presumably as a result of defective DDR his si associated with inductions of histone 2A.X phosphorylation that is canonically associated with DNA damage. PfSR1 was also shown to co-localize with the phosphorylated form of H2A.X after X-ray irradiation, which indicates a recruitment of PfSR1 to the sites of DNA damage. Crucially, exposure of P. falciparum to artemisinin, the key compound of the current malaria chemotherapeutics that can also cause DNA damage had similar effect on the PfSR1-DDR-related phenotypes as those induced by X-ray irradiation.
Overall, I find the manuscript well presented and the results well substantiated. Given that artemisinin resistance is currently a main issue that hinders the world-wide elimination program, this manuscript is also of high importance. I would happily support its publication in JCS.

Comments for the author

I have only a few minor concerns regarding the results and their interpretations. I believe addressing these will improve the manuscript and disperse some doubts about its relevance.

1. In the initial immunoprecipitation experiment the authors found considerably stronger interactivity of PfSR1 in the early (rings) stage (121 proteins) compared to the late stage (schizonts) with only 28 proteins and only 4 being common. This is somewhat counterintuitive as DDR is expected to be much more active in the schizont stages when most of the DNA synthesis occurs. This was also shown by the authors in this manuscript, when PfSR1 was recruited to multiple DDR loci in the schizont stages. It would be helpful to provide more comments and information on the PfSR1 connectivity relative to the developmental stage distribution. Is the DDR associating of PfSR1 ring specific or does it also happen in schizonts? Also how much is the immunoprecipitation result affected by PfSR1 abundance and an expression pattern given that it was expressed episomally, not under the control of its own promoter?

2. In the figure 2C the authors showed slower growth rate of the PfSR1 knockdown that is mediated by exposure to 5mM of GlcN. However, there is no control with a wild type parasites (either the parental NF54 or (better) “empty vector”) in the presence of 5mM of GlcN. I believe such control should be provided if the slow growth phenotype is to be presented.

3. In Fig 3D the author demonstrated that continuous disruption of PfSR1 by 5m GlcN leads to increase incidence of “spontaneous” DNA damage that is evident by increased levels of H2A.X phosphorylation in about a week. In my view this result could be stranger if the authors could also show this by the TUNEL assay similarly to Fig 3B. It might be also informative to see if the main difference comes from the early ring stages when the PfSR1 is presumably more connected to the DDR machinery of the older schizont stages during DNA replication.

4. In figure 4 the authors demonstrated the recruitment of PfSR1 to the sites of DNA damage by co-immunostaining with the phosphorylated H2A.X and RAD51 in parasites after X-ray irradiation. Similarly histone isolation and H2A.X immunoprecipitation showed a strong presence of PfSR1 in material obtained from irradiated parasites. In my view this experiment is missing a crucial negative control done with parasites that were NOT irradiated and little or no DDR is occurring. Where is PfSR1 located under normal conditions?

Does the the DDR-loci localization represent shuttling from other compartments? Is PfSR1 associated (or not) with histones even under normal conditions?

5. In figure 6 the authors demonstrated a similarity of the PfSR1 knock down parasites growth characteristic under the X-ray irradiation to those induced by artemisinins. This certainly makes a compelling case for DNA damage involvement in artemisinin mode of action and DDR is a putative resistance mechanism.
However, artemisinin is also known to affect other biological functions some of these being also associated with PfSR1 binding partners such as ribosomal biogenesis. Can the authors exclude the

© 2021. Published by The Company of Biologists under the terms of the Creative Commons Attribution License (https://creativecommons.org/licenses/by/4.0/).

fact that the PfSR1 activity in the artemisinins-induced phenotypes is not caused by that rather than DDR? If not they should at least comment on it.

First revision

Author response to reviewers' comments

Dear Dr. Carmo Fonseca,

We have revised our recent manuscript An SR protein is essential for activating DNA repair in malaria parasites according to the reviewers comments. Please find below a summary of the changes. On behalf of all authors I would like to thank the reviewers for their thorough reading of the manuscript. Their careful criticism and constructive suggestions have greatly contributed to the improvement of our paper.

The major improvements include the addition of better controlled experiments demonstrating the necessity of *Pf*SR1 for DNA repair and for the accumulation of *Pf*RAD51 in the parasite's nucleus. We have also included data from a nuclear fractionation experiment before and after parasite irradiation that indicated that the ratio of chromatin bound *Pf*SR1 is elevated following parasite irradiation. This data is now presented in the revised figure 5, as well as in the supplementary figures S5 & S6 that include imaging panels of PfSR1 association with damaged chromatin and PfRAD51 before and after irradiation.

In addition, we have revised Figures 1 and 2 and made revisions to the text according to the reviewers' suggestions. All changes in the text are marked in red.

We are happy that both reviewers found the paper interesting and important and thought it should be published. We hope that these revisions address their concerns.

Reviewer 1 Comments for the Author:
I recommend revising the paper mostly in the presentation of the data included.

We thank the reviewers for her/his valuable suggestions and made the recommended changes in data presentation as well as adding significant new data (see below). We believe these changes significantly improved our manuscript.

The paper would be improved by re-focusing the intro and conclusion on DNA repair and providing a hypothesis on the potential roles that SR1 may play in the parasite DDR within the context of what is known in higher eukaryotes. They reference review papers on the topic but do not explicitly provide any ideas on mechanism based on the literature and/or the data they present.

We have included a more detailed explicit discussion on the current knowledge on the direct implications of eukaryotic splicing factors in DDR. In addition, we have included our new finding associating PfSR1 with damaged chromatin together with PfSR1 dependent accumulation of PfRAD51 in the nucleus with DNA repair (see below). This data supports our hypothesis that PfSR1 plays a direct role in the repair machinery in *P. falciparum*. We carefully discuss this hypothesis in the discussion section as the mechanisms by which SR proteins are involved in DDR in other eukaryotes are still elusive.

Parts of the abstract should be re-phrased. They state that PfSR1 is "required for activating DNA machinery" but do not have evidence of this, merely that the knockdown is delayed in its ability to repair DNA damage.

The abstract was shortened and this statement was taken out.

They also state that "loss of PfSR1 does not impair parasite viability" yet in figure 1D and

© 2021. Published by The Company of Biologists under the terms of the Creative Commons Attribution License (https://creativecommons.org/licenses/by/4.0/).

supplement 4 they demonstrate a slow growth phenotype which is likely multifactorial. In addition, they focus their discussion on replicative stress, yet the late stage interactome indicates less impact of SR1 at this stage when the parasite is actively replicating its DNA and the growth delay appears to be minimal (though hard to tell from supplementary fig 4) and is likely multifactorial.

The reviewer is absolutely correct. While the loss of PfSR1 does not impair parasite viability, it causes a slight delay in the growth rate of parasite populations in culture. We agree that this delay in population growth is likely multifactorial. We tried to emphasized and make it clearer in the text. Following this (and additional comment) Fig. 2D was replaced with the data including the right control (both lines on GlcN) as suggested by both reviewers. We also provide a better explanation and reference to the figure legends that will help readers to better understand Fig. S4.

We agree that the late stage interactome indicated less impact of PfSR1 at that stage. However, the pull-downs of PfSR1 interacting proteins was performed on parasites growing under normal conditions as an initial screen for additional biological function of PfSR1. This screen pointed us towards the possible role of PfSR1 in DDR which led to the main hypothesis of the paper. This hypothesis was further functionally tested by creating the inducible k/d system and led to the discovery of the role of PfSR1 in DDR which is the main focus of the current manuscript. Elucidating the entire machinery by which PfSR1 is involved in the repair of damage from different sources is an excellent idea for future directions, but is beyond the scope of the current paper.

I would recommend restructuring Figure 1. The small number of proteins at the ring stage make the late stage pie chart minimally informative. As this is a DNA repair focused paper, a table of the DNA repair proteins identified is much more meaningful and those proteins should be part of their discussion.
In addition, the gene annotations in the interaction networks are outdated and should be updated to match those used in the supplementary table and current databases.

We have restructured Fig. 1 according to the reviewer's suggestion and included a table containing the proteins implicated in DDR which were pulled down with PfSR1 and added them to the discussion, as suggested. In addition, we have included the updated accession numbers to the proteins presented in Fig. 2C.

They present data that Rad51 and PfSR1 are both recruited to sites of DNA damage post irradiation. If they demonstrated that Rad51 is not recruited to sites of damage post irradiation in the knockdown, then that would support the hypothesis that PfSR1 is required to activate the DNA repair machinery thus providing insight into the mechanism for the persistent DNA damage in the PfSR1 knockdown.

We agree and we thank the reviewer for this comment. Following this constructive critic, and in order to provide insight into the mechanism for the persistent DNA damage in the PfSR1 knockdown, we have performed a better controlled set of experiments. Our new data provide evidence that prior to exposure to the source of damage PfRad51 does not accumulate in the nucleus and following parasite exposure to irradiation it accumulates in the nucleus and co-localizes with PfSR1. Moreover, we also demonstrate that in the absence of PfSR1 expression PfRad51 does not accumulate in the nucleus even after exposure to irradiation.
Furthermore, we show that this co-localization is associate with repair, when PfSR1 is expressed, PfRad51 accumulate in the nucleus parallel to the decrease in the level of γ- PfH2A indicating that the parasite activated DDR. However, in the absence of PfSR1 expression, when the levels of nuclear PfRad51 do not increase following irradiation - the levels of γ-PfH2A do not decrease…indicating that the parasite was unable to activate DDR. These data are now included in the new supplementary figures S5 and S6, which demonstrate the localizations and in the revised Figure 5.

It appears they are using DHA merely as a mutagen, albeit a potentially clinically relevant mutagen, and are not proposing that PfSR1 contributes to DHA mechanism of action or resistance. This should be clarified, especially in light of the UPS proteins identified in the interactome studies. The UPS has been identified as key to ART resistance and is equally or more likely to impact ART survival and this should at least be discussed.

© 2021. Published by The Company of Biologists under the terms of the Creative Commons Attribution License (https://creativecommons.org/licenses/by/4.0/).

We agree and added this point to the discussion.

Minor comments:
-In the intro and discussion they mention only a subset of DNA repair pathways present in the parasite, ie only MMR for non DSB repair. They should be comprehensive in their discussion or state they are only focusing on DNA DSBs.

We added a statement that we focus on DNA DSB.

-Supplemental fig 4 would be more informative transformed into a growth line

We apologize for this confusion. Fig. S4 is indeed a long term growth curve that includes the required "cut downs" when the culture reach high parasitemia, in order to avoid parasite death. We have included a reference and better explanation to the figure's legends.

Reviewer 2
Overall, I find the manuscript well presented and the results well substantiated. Given that artemisinin resistance is currently a main issue that hinders the world-wide elimination program, this manuscript is also of high importance. I would happily support its publication in JCS.

We thank the reviewer for his/her warm words and support of our paper.

Reviewer 2 Comments for the Author:
I have only a few minor concerns regarding the results and their interpretations. I believe addressing these will improve the manuscript and disperse some doubts about its relevance.

1. In the initial immunoprecipitation experiment the authors found considerably stronger interactivity of PfSR1 in the early (rings) stage (121 proteins) compared to the late stage (schizonts) with only 28 proteins and only 4 being common. This is somewhat counterintuitive as DDR is expected to be much more active in the schizont stages when most of the DNA synthesis occurs. This was also shown by the authors in this manuscript, when PfSR1 was recruited to multiple DDR loci in the schizont stages. It would be helpful to provide more comments and information on the PfSR1 connectivity relative to the developmental stage distribution. Is the DDR associating of PfSR1 ring specific or does it also happen in schizonts? Also how much is the immunoprecipitation result affected by PfSR1 abundance and an expression pattern given that it was expressed episomally, not under the control of its own promoter?

The reviewer points are correct. PfSR1 interacting proteins were pulled down using a Halo-tag-based episomal system under normal growth conditions, which provided us with the initial indication for the possible role of PfSR1 in DDR. This hypothesis was further functionally tested by various experiments in the rest of the paper, which were done on an endogenously tagged inducible knock down system of PfSR1. The endogenous knock down line enabled us to demonstrate the novel role for PfSR1 in DDR and provided some hints towards possible mechanisms involved. Nonetheless, we agree that deciphering the entire machinery is an important future direction.

We also agree (and stated so in the paper) that that replicative errors during schizogony could cause significant damage to the parasite genome that must ensure the integrity of its genome during this process. However, in our hands we find that replicating stages of Plasmodium are extremely resilient to various sources of damage including very high doses of irradiation and thus, no distinct phenotype is observed in late stages and it is difficult to determine PfSR1 involvement in DNA repair at that stage even though we show that it is associated with the site of damage and PfRad51. Therefore, at this point we focused our study on ring stage parasites which are sensitive to sources of DNA damage to demonstrate the involvement of PfSR1 in DDR.

Fig. 1 was revised according to the reviewers' suggestion and focus on PfSR1 interactome at early stages. We further emphasized in the discussion that the Pull-down data we presented was obtained by an episomal system.

2. In the figure 2C the authors showed slower growth rate of the PfSR1 knockdown that is

© 2021. Published by The Company of Biologists under the terms of the Creative Commons Attribution License (https://creativecommons.org/licenses/by/4.0/).

mediated by exposure to 5mM of GlcN. However, there is no control with a wild type parasites (either the parental NF54 or (better) “empty vector”) in the presence of 5mM of GlcN. I believe such control should be provided if the slow growth phenotype is to be presented.

The reviewer’s point is absolutely right. We had the data and included it in the revised figure 2D according to this suggestion.

3. In Fig 3D the author demonstrated that continuous disruption of PfSR1 by 5m GlcN leads to increase incidence of “spontaneous” DNA damage that is evident by increased levels of H2A.X phosphorylation in about a week. In my view this result could be stranger if the authors could also show this by the TUNEL assay similarly to Fig 3B. It might be also informative to see if the main difference comes from the early ring stages when the PfSR1 is presumably more connected to the DDR machinery of the older schizont stages during DNA replication.

Following this comment, we performed TUNEL assay on the PfSR1 k/d line continuously growing on GlcN. However, despite several attempts we were unable to get obvious differential signal as the one in which we were able to obtained by TUNEL done on parasites which were irradiated (Fig. 3B). It is likely that exposure to irradiation cause more substantial DNA damage than PfSR1 knock-down that enable good detection by TUNEL (in comparison to control parasite that are not irradiated) while the damage caused by long term PfSR1 k/d could only be detected by Western blot.

4. In figure 4 the authors demonstrated the recruitment of PfSR1 to the sites of DNA damage by co-immunostaining with the phosphorylated H2A.X and RAD51 in parasites after X-ray irradiation. Similarly histone isolation and H2A.X immunoprecipitation showed a strong presence of PfSR1 in material obtained from irradiated parasites. In my view this experiment is missing a crucial negative control done with parasites that were NOT irradiated and little or no DDR is occurring. Where is PfSR1 located under normal conditions? Does the the DDR-loci localization represent shuttling from other compartments? Is PfSR1 associated (or not) with histones even under normal conditions?

We agree with this comment which raised by both reviewers. We have included new data in the revised Fig. 5C and two supplementary figures S5 and S6 that demonstrate the localization γ-PfH2A and PfRad51 before and after irradiation in parasites that express PfSR1 and those in which PfSR1 is knocked-down (see also response to reviewer #1). This set of experiments demonstrated that PfRad51 co-localization with PfSR1 is associate with repair. When PfSR1 is expressed, PfRad51 accumulate in the nucleus in parallel to the decrease in the level of γ-PfH2A indicating that the parasite activated DDR. However, in the absence of PfSR1 expression the levels of nuclear PfRad51 do not increase following irradiation and the levels γ-PfH2A do not decrease…indicating that the parasite was unable to activate DDR.
In addition, we have performed nuclear fractionation before and after parasite irradiation. This experiment indicated that the ratio between chromatin and nucloplasmic PfSR1 is elevated following parasite irradiation. This Data is included in the revised fig. 5.

We believe that by addressing this constructive critic we significantly improved our paper and thank the reviewers for their contribution.

5. In figure 6 the authors demonstrated a similarity of the PfSR1 knock down parasites growth characteristic under the X-ray irradiation to those induced by artemisinins. This certainly makes a compelling case for DNA damage involvement in artemisinin mode of action and DDR is a putative resistance mechanism. However, artemisinin is also known to affect other biological functions some of these being also associated with PfSR1 binding partners such as ribosomal biogenesis. Can the authors exclude the fact that the PfSR1 activity in the artemisinins-induced phenotypes is not caused by that rather than DDR? If not they should at least comment on it.

We added the possibility to the discussion as suggested.

© 2021. Published by The Company of Biologists under the terms of the Creative Commons Attribution License (https://creativecommons.org/licenses/by/4.0/).

Second decision letter

MS ID#: JOCES/2021/258572

MS TITLE: An SR protein is essential for activating DNA repair in malaria parasites

AUTHORS: Manish Goyal, Brajesh Kumar Singh, Karina Simantov, Yotam Kaufman, Shiri Eshar, and Ron Dzikowski

ARTICLE TYPE: Research Article

I am happy to tell you that your manuscript has been accepted for publication in Journal of Cell Science, pending standard ethics checks.

Reviewer 1

Advance summary and potential significance to field

This revised manuscript is much improved, and the authors appear to have fully addressed both reviewer's concerns. The new data strengthens their hypothesis considerably and provides a working model of the role of PfSR1 in the initiation of the DDR in the malaria parasite. In this way the paper makes significant and novel contributions to the cell biology of P falciparum and DNA damage response in eukaryotes in general.

Comments for the author

The paper is suitable for publication as it stands.

Reviewer 2

Advance summary and potential significance to field

This manuscript summarizes a study of the DNA damage repair mechanism in the human malaria parasites Plasmodium falciparum. In particular, it was found that a highly conserved RNA splicing factor, PfSR1, plays an unexpected and crucial role in the DNA repair machinery, which represents a highly unique example of evolutionary functional diversion in the malaria parasite species. This possibly mitigates other anomalies such as the lack of the NHEJ pathway for repairing DNA double strand breaks that might have “forced” this diversion. Here the authors use an inducible and reversible system to manipulate PfSR1 expression, and show that this protein is recruited to foci of DNA damage. Loss of PfSR1 is essential for parasites recovery from exposure to artemisinin; the first line antimalarial drug, which was previously shown toe cause DNA damage.
These findings provide key insights into the evolution of DNA repair pathways in malaria parasites as well as the parasite's ability to recover from antimalarial treatment.

Comments for the author

All concerns were addressed appropriately. I recommend this manuscript for publication.

© 2021. Published by The Company of Biologists under the terms of the Creative Commons Attribution License (https://creativecommons.org/licenses/by/4.0/).
